# Development of a Well-Characterized Rhesus Macaque Model of Ebola Virus Disease for Support of Product Development

**DOI:** 10.3390/microorganisms9030489

**Published:** 2021-02-26

**Authors:** Kendra J. Alfson, Yenny Goez-Gazi, Michal Gazi, Hilary Staples, Marc Mattix, Anysha Ticer, Benjamin Klaffke, Kaylee Stanfield, Priscilla Escareno, Patrick Keiser, Anthony Griffiths, Ying-Liang Chou, Nancy Niemuth, Gabe T. Meister, Chris M. Cirimotich, Ricardo Carrion

**Affiliations:** 1 Disease Intervention and Prevention Program, Texas Biomedical Research Institute, 8715 W. Military Dr., San Antonio, TX 78227, USA; kalfson@txbiomed.org (K.J.A.); ygoez@txbiomed.org (Y.G.-G.); mgazi@txbiomed.org (M.G.); hstaples@txbiomed.org (H.S.); aticer@txbiomed.org (A.T.); bklaffke@txbiomed.org (B.K.); ks1341@gmail.com (K.S.); pescareno@txbiomed.org (P.E.); pkeiser@bu.edu (P.K.); ahgriff@bu.edu (A.G.); 2 Nonclinical Pathology Services, LLC, 3000 Stonebrooke Ln, Medina, OH 44256, USA; rwpath@gmail.com; 3 Battelle Biomedical Research Center (BBRC), 1425 Plain City Georgesville Road, West Jefferson, OH 43162, USA; chouyl@battelle.org (Y.-L.C.); niemuth@battelle.org (N.N.); meisterg@battelle.org (G.T.M.); cirimotichc@battelle.org (C.M.C.)

**Keywords:** Ebola virus, rhesus macaque, animal model, FDA Animal Rule, natural history

## Abstract

Ebola virus (EBOV) is a negative-sense RNA virus that can infect humans and nonhuman primates with severe health consequences. Development of countermeasures requires a thorough understanding of the interaction between host and pathogen, and the course of disease. The goal of this study was to further characterize EBOV disease in a uniformly lethal rhesus macaque model, in order to support development of a well-characterized model following rigorous quality standards. Rhesus macaques were intramuscularly exposed to EBOV and one group was euthanized at predetermined time points to characterize progression of disease. A second group was not scheduled for euthanasia in order to analyze survival, changes in physiology, clinical pathology, terminal pathology, and telemetry kinetics. On day 3, sporadic viremia was observed and pathological evidence was noted in lymph nodes. By day 5, viremia was detected in all EBOV exposed animals and pathological evidence was noted in the liver, spleen, and gastrointestinal tissues. These data support the notion that EBOV infection in rhesus macaques is a rapid systemic disease similar to infection in humans, under a compressed time scale. Biomarkers that correlated with disease progression at the earliest stages of infection were observed thereby identifying potential “trigger-to-treat” for use in therapeutic studies.

## 1. Introduction

*Ebolavirus* and *Marburgvirus*, members of the family *Filoviridae*, are negative-sense, single-stranded, RNA viruses that are known to infect humans and nonhuman primates (NHPs) with severe health consequences, including death. Filovirus infections have resulted in case fatality rates of up to 90% in humans [1]. Members of the *Ebolavirus* and *Marburgvirus* generas can cause Ebola virus disease (EVD) and Marburg virus disease (MVD)—previously referred to as Ebola or Marburg hemorrhagic fever [2]—with death often occurring within 7 to 10 days post-exposure [1,3,4]. Ebola virus disease presents as an acute febrile syndrome manifested by an abrupt fever, nausea, vomiting, diarrhea, maculopapular rash, malaise, prostration, generalized signs of increased vascular permeability, coagulation abnormalities, and dysregulation of the innate immune response. Much of the disease appears to be caused by dysregulation of innate immune responses to the infection and by replication of virus in vascular endothelial cells, which induces death of host cells and destruction of the endothelial barrier, though mortality can result from numerous factors [1,4,5,6,7]. Filoviruses can be spread by direct or indirect contact with infected blood, organs, and body fluids from infected humans or animals [7,8]. Infection with less than one plaque forming unit (PFU) is reported to be sufficient to cause disease in NHPs [9,10]. The reservoir for filoviruses has not yet been definitively identified, but is thought to be bats [11,12]. As the cause of severe human disease, filoviruses continue to be of concern as both a source of natural infections, and also as possible agents of bioterrorism. 

The sporadic nature of filovirus outbreaks, the high mortality associated with infection, and the inability to ethically perform exposure studies in humans means the evaluation of filovirus countermeasures may necessitate compliance with the “FDA Animal Rule” (see 21 CFR 314.60 for drugs or 21 CFR 601.90 for biological products) for efficacy evaluation [13,14]. Confidence on the applicability of an animal model relies on, among other things: demonstrating that disease in the model is well-understood, the model can be considered well-characterized and adequate for demonstration of efficacy, and efficacy endpoints are clearly related to the desired outcome in humans (e.g., improved survival or reductions in major morbidity) [13,14,15]. 

As such, it is desirable to develop well-characterized animal models, utilizing studies that meet FDA standards of being adequate and well controlled. This involves characterizing a specific exposure agent, via a specific route at a specific target dose in a specific animal species and disease outcomes must correspond to critical aspects of the human disease. Such models can be used for efficacy testing for multiple investigational vaccines and therapeutics, which is beneficial to medical countermeasure development through reduced financial expenditures, timelines, animals, and resources required for each product. Currently, many different animal models of filovirus infection and disease exist but many are poorly characterized, not well understood, or do not closely recapitulate human disease (reviewed in [16,17,18]). This is a problem for efficacy testing under the animal rule, and a detriment to performing basic foundational science. Macaques (rhesus macaque, *Macaca mulatta* and cynomolgus macaque, *Macaca fascicularis*) are the most frequently used NHP models. Wild type filoviruses are lethal in these animals and they exhibit symptoms similar to those seen in humans ([18,19]). However, research design varies between facilities conducting these experiments and a standard model has not been chosen, let alone qualified.

The primary objective of this study was to further characterize the natural history of Ebola virus (EBOV) in a uniformly lethal rhesus macaque model. Many of the parameters measured in this study have also been measured in previous studies ([17,18,20,21,22]). However, we aim to expand upon previous descriptions of the model to confirm reproducibility, and characterize disease progression through the use of planned serial euthanasia starting at early timepoints. This study consisted of two arms, one group of animals was euthanized at pre-determined timepoints beginning three days post exposure to characterize disease progression by analyzing viremia, clinical pathology, immunology, and histopathology. Additionally, for the analysis of survival, changes in physiology, clinical pathology, and telemetry kinetics, four animals were not scheduled for serial euthanasia. A final group of animals was mock exposed to phosphate buffered saline solution (PBS) as controls. Blood was collected on pre-determined days 0, 3, 4, 5, and 6 relative to EBOV exposure. Time to death, presence or absence and degree of viremia, body weights and temperatures, clinical observations, changes in clinical pathology (hematology, clinical chemistry, and coagulation), gross necropsy/histopathology, and biomarkers that could have the potential to serve as triggers of treatment initiation during therapeutic studies were assessed.

## 2. Materials and Methods

### 2.1. Ethics Statement

Animal research was conducted under an Institutional Animal Care and Use Committee (IACUC)-approved protocol (IACUC number 1604MM; approved August 25, 2017) in compliance with the Animal Welfare Act, Public Health Service (PHS) policy, and federal regulations related to experiments involving animals. Texas Biomedical Research Institute follows the National Research Council Guide for the Care and Use of Laboratory Animals [23], and is accredited by the Association for Assessment and Accreditation of Laboratory Animal Care. Study veterinarians approved all euthanasia, and euthanasia was performed using an intravenous overdose of sodium pentobarbital. Previously developed euthanasia criteria were adhered to in order to minimize pain and distress.

### 2.2. Animal Care

Twelve male and 12 female rhesus macaques (*Macaca mulatta,* Chinese origin), 3 to 7 years of age and weighing between 4.1–7 kg at EBOV challenge were obtained from Envigo (former Covance; Alice, TX, USA) and used in this study. Prior to EBOV exposure, animals were housed individually in standard cages and acclimated to an Animal Biosafety Level 4 (ABSL-4) laboratory. The NHPs were experimentally naïve and found to be seronegative for Simian Immunodeficiency Virus (SIV), Simian T-Lymphotropic Virus-1 (STLV-1), Simian Varicella Virus (SVV), and *Macacine herpesvirus* 1 (Herpes B virus), negative for Simian Retrovirus (SRV1 and SRV2) by polymerase chain reaction (PCR), negative for *Trypanosoma cruzi* (PCR and serology), and free from active infections with *Salmonella* and *Shigella* bacteria. Animals were also tested and verified negative for tuberculosis and antibody-negative for Ebola Reston (screened by Virus Reference Laboratory, San Antonio, TX, USA). Additionally, NHPs were tested at Texas Biomed to ensure seronegativity to EBOV, Sudan virus, or Marburg virus glycoprotein.

Commercial primate diet from Purina Mills (5LEO 15% Monkey Diet) was provided at least twice daily, except when animals were fasted for sedation. Water from the institutional watering system was available ad libitum. Animals were provided with perches and manipulable toys as inanimate enrichment, and food enrichment at least five times per week. The excreta pans were under the cages; cage flooring and room floors were cleaned daily. The targeted environmental and photoperiod conditions were: temperature of 74 °F ± 10 °F, humidity of approximately 30% to 70%, and 12 h on/12 h off light cycle.

Animals were evaluated by a study veterinarian after arrival at Texas Biomed to confirm health prior to study initiation. Animals were acclimated to ABSL-4 laboratory conditions for 7 days prior to EBOV exposure and were observed twice daily during this time. Clinical observations involved evaluating each animal for 13 different physical parameters and assigning a numerical score to each parameter based on severity (Table 1). Scores were then added up to achieve a total clinical score, which was reported to the study veterinarian when above a score of 3. After exposure, animals were observed at least twice daily and more frequently as clinical signs warranted, until the end of the project; a clinical score of 4 to 7 in any animal resulted in all animals being observed at least three times per day and a clinical score greater than 7 in any animal resulted in all animals being observed at least four times a day. All observations were performed at least approximately 6 h apart.

Euthanasia criteria were as follows (previously described, [24]): animals were humanely euthanized as soon as any of the following occurred—(1) total clinical score greater than or equal to 15; (2) prostrate but able to rise if stimulated with greater than 5 degree F temperature change from baseline; or (3) prostrate but able to rise if stimulated and any two of the following were true (from most recent blood draw): alanine aminotransferase (ALT) greater than 200 U/L; Alkaline phosphatase (ALP) greater than 1100 U/L; Gamma-glutamyl transferase (GGT) greater than 170 U/L; blood urea nitrogen (BUN) greater than 30 mg/dL; Albumin (ALB) less than 3.0 g/dL. 

### 2.3. Cells and Virus

Vero E6 cells (NR-596; BEI resources, Manassas, VA, USA) were used in this study. Cells were maintained in Minimum Essential Media (MEM; Gibco, Grand Island, NY, USA) supplemented with 2 mM/L-glutamine (Gibco, Grand Island, NY, USA) and 1 mM sodium pyruvate (Gibco, Grand Island, NY, USA) and 10% heat-inactivated fetal bovine serum (FBS; Gibco, Grand Island, NY, USA) at 37 °C with 5% CO_2_ [9].

A second-cell culture passage (P2) of EBOV (Kikwit variant) was supplied by Dr. Tom Ksiazek (at National Institute of Allergies and Infectious Diseases (NIAID’s) World Reference Center for Emerging Viruses and Arboviruses (WRCEVA) at the University of Texas Medical Branch (UTMB’s) Health Galveston National Laboratory) in 2012. The stock virus was propagated from this P2 EBOV (Ebola virus H.sapiens-tc/COD/1995/Kikwit-9510621, species *Zaire ebolavirus*) via passaging one time on Vero E6 cells at a multiplicity of infection of 0.001, to generate a P3 virus stock for use in NHP experiments [10]. The P3 viral stock was characterized for infectious virus content by plaque assay, purity, and microbial and toxin contamination, was confirmed to be wild-type Ebola virus by deep sequencing, and was assigned Lot No. 201209171.

### 2.4. Experimental Exposure of NHPs to EBOV

The study was performed in two iterations with each iteration comprised of 10 animals at ABSL-4 and two mock-infected animals housed at animal biosafety level 2 (ABSL-2); the execution of each study iteration was identical and the data from both iterations have been pooled.

NHPs were randomly assigned to EBOV-exposure or mock-exposure control groups, as well as randomly selected for scheduled euthanasia time points. NHPs were sedated and exposed in ascending order as determined by their animal tattoo number and the order and time of exposure was recorded. Stata (version 12.1) was used to randomize the animals; random seed values were selected using R (version 2.14.1).

Twenty NHPs were exposed to a targeted dose of 1000 PFU of Ebola virus and four control animals (housed at ABSL-2) were mock exposed using sterile PBS. Prior to exposure, each animal was sedated by an IM injection of tiletamine hydrochloride/zolazepam hydrochloride (Telazol, 2–8 mg/kg of body weight; Fort Dodge Laboratories, Fort Dodge, IA). A total of 0.5 mL of exposure material was administered to each animal via intramuscular (IM) injection in the right deltoid muscle of the arm. The administration site was recorded. Following exposure, each NHP was taken back to its home cage and observed until it had recovered from anesthesia (sternal recumbency/ability to maintain an upright posture). Animals were sedated on scheduled time points and monitoring of rectal temperatures, body weight, and blood samples for clinical pathology and viremia was performed on pre-determined days 7, 0, 3, 4, 5, 6, scheduled euthanasia, or at time of euthanasia in moribund animals. A total of four EBOV exposed animals were euthanized each day as scheduled on days 3, 4, 5, 6 post-exposure. The remaining four EBOV exposed animals died or were euthanized due to moribundity on days 7 to 10 post exposure. The mock exposure control animals were euthanized as scheduled on day 6 post-exposure.

### 2.5. Preparation and Verification of Exposure Dose

EBOV stock was diluted via two consecutive 1:10 dilutions to a target dose of 2000 PFU/mL in sterile phosphate buffered saline (PBS) and the preparation was recorded on an exposure dose preparation sheet. Following exposure agent preparation, an aliquot was removed for determination of titer. After the last animal was exposed, the vial containing the exposure material was used for post-exposure titer determination. Titer of exposure materials was determined via Neutral Red Agarose Overlay (NRAO) plaque assay (as previously described [25]).

### 2.6. Determination of Viral Titers

Viral load was determined from plaque assays performed on serum collected at pre-determined study days 0, 3, 4, 5, 6, (relative to exposure date), at scheduled euthanasia, and at moribund euthanasia. At necropsy, samples of tissues were aseptically collected from each animal. Mock exposed control animals had blood samples collected on the same study day schedule to obtain serum. Aliquots of serum samples and tissue samples were stored at –80 ± 10 ºC and samples taken from EBOV exposed animals were subsequently processed for viral load determination by NRAO plaque assay (as previously described, (18)). Briefly, Vero E6 cells in Dulbecco’s Modified Eagle Media (DMEM; Gibco, Grand Island, NY, USA) containing 1 mM sodium pyruvate (Gibco, Grand Island, NY, USA) (henceforth referred to as normal growth media) with 10% heat-inactivated fetal calf serum (FCS) were seeded at a density of 7.5 to 9 × 10^5^ cells per well in 6-well tissue culture plates. On the day of the assay, serial dilutions of virus were prepared in normal growth medium containing 2% FCS and plated in the corresponding well of a 6-well plate. After a 1 h incubation at 37 °C with 5% CO_2_, media were removed from the wells and a 2 mL primary overlay was added (1% Seakem agarose (Lonza, Morristown, NJ, USA) mixed 1:1 with 2× Eagle’s MEM (EMEM; Lonza, Morristown, NJ, USA) containing 4 mM L-glutamine, 2 mM sodium pyruvate, and 4% FCS). After seven days, 2 mL of secondary overlay was added to each well (primary overlay formulation also containing 4% final concentration neutral red solution (Gibco, Grand Island, NY, USA)). Cells were incubated for one day, and then plates were scanned for manual plaque counting to determine a final titer.

### 2.7. Determination of Viral Genome Copies

Viral RNA was quantified from serum as well as tissue samples via quantitative reverse transcription-PCR (qRT-PCR) using Applied Biosystems Quant Studio 3 real-time PCR instrument (Life Technologies Corporation, Carlsbad, CA, USA). Serum was inactivated using RNAbee (Tel-Test, Friendswood, TX, USA) and RNA was isolated according to the manufacturer’s instructions. One-step qRT-PCR was performed using RNA UltraSense One-Step Quantitative RT-PCR System (Invitrogen, Carlsbad, CA, USA), and primers and probe specifically designed to detect a region of the EBOV glycoprotein gene [26]; this One-Step method and primers will detect all RNA corresponding to the Ebola virus GP gene (including antigenome and mRNA).

### 2.8. Determination of Body Temperature and Activity via Telemetry

Body temperature data was collected from M00 telemeter implants. Animals were implanted with Data Sciences International (DSI, New Brighton, MN, USA) M00 telemeters. Upon study completion, data collected from the implants were uploaded to an online enterprise content management platform called Box (Redwood City, CA, USA) for access by DSI personnel. The hourly averages were compared to the baseline established before exposure. Data was only processed if the signals were of sufficient quality to be analyzed. Data were omitted in the case of signal drop out and/or non-physiological values. Parameters evaluated were: Temperature, T-mean, Activity, and Mean activity.

### 2.9. Hematology, Blood Chemistry and Coagulation Tests

Whole blood was collected at pre-determined study days 7, 0, 3, 4, 5, 6, at scheduled euthanasia, and at moribund euthanasia. Whole blood was collected into tubes containing Ethylenediaminetetraacetic acid (EDTA) for Complete blood Counts (CBC) analysis and clinical chemistry. CBC was performed using a Procyte Dx Hematology Analyzer (IDEXX laboratories, Westbrook, Maine, USA) and the parameters analyzed included white blood cell counts, neutrophil counts and percent, lymphocytes counts and percent, monocytes counts and percent, red blood cell counts, percent hematocrit, reticulocyte counts and percent, platelet counts, and percent platelet. Clinical chemistry was performed using the mammalian liver enzyme profile rotor on a Vet Scan analyzer (Abaxis, Inc., Union City, CA, USA). Coagulation times were determined on whole blood with no additives using an IDEXX Coag Dx Analyzer (IDEXX Laboratories, Westbrook, Maine, USA). Parameters were evaluated against reference ranges established by Texas Biomed clinical pathology derived from a cohort of clinically healthy macaques.

### 2.10. Cytokines and Chemokines Analysis

Serum collected on pre-determined study days 0, 3, 4, 5, 6, and at euthanasia were analyzed for the profiles of specific soluble mediators (cytokines and chemokines) using the MILLIPLEX MAP Non-Human Primate Cytokine Magnetic Bead Panel: Immunology Multiplex Assay (PRCYTOMAG-40K, Millipore Corporation, Billerica, MA, USA), along with Luminex xMAP detection, to examine the cytokine profiles in the ABSL-4 at Texas Biomed. Analytes assayed included the following: G-CSF, GM-CSF, IFN-γ, IL-1ra, IL-1β, IL-2, IL-4, IL-5, IL-6, IL-8, IL-10, IL-12/23 (p40), IL-13, IL-15, IL-17, IL-18, MCP-1, MIP-1α, MIP-1β, sCD40L, TGF-α, TNF-α, VEG. This kit allows for simultaneous analysis of multiple cytokine and chemokine biomarkers with bead-based multiplex assays using the Luminex technology in non-human primate serum. Briefly, magnetic microspheres coated with specific capture antibodies are coupled to serum samples, which are then biotinylated after an antibody is added. The coupled reaction is then captured by a streptavidin-PE conjugate (reporter molecule) and the Luminex^®^ analyzer (Austin, TX, USA) acquires and quantifies based on fluorescent reporters. Multiplex assay analysis was done with the Milliplex Analyst xPonent^®^ 4.2 software for MAGPIX^®^. Values outside the upper or lower limits (out of range values) of the standard curve stablished by the manufacturer were considered maximum or minimum values, respectively.

### 2.11. D-Dimer Assay

A commercial kit (Aserachrom D-Di; Diagnostica Stago, Parsippany, NJ, USA) was used to quantitate the concentrations of d-dimers, a product of plasmin digestion of cross-linked fibrin, using an assay targeting the fibrin degradation product in plasma collected on pre-determined study days 0, 3, 4, 5, 6, and at euthanasia, following manufacturer instructions. Briefly, the assay was performed using a 96-well plate coated with a mouse monoclonal anti-human D-Di antibody. D-Di in diluted plasma samples is captured by the immobilized antibody and sandwiched with a peroxidase-rabbit anti–fragment D antibody which is colorimetrically detected using TMB substrate. Reaction volumes used throughout the assay were 200 µL. Sulfuric acid was used to terminate color development. A Bio-Rad iMark^®^ Microplate reader (Bio-Rad, Hercules, California, USA) was used to obtain optical density readings. The manufacturer provided calibrator reagent was used to generate standard curves, that were used to calculate D-Di concentrations.

### 2.12. Soluble Glycoprotein (sGP) Analysis

For the quantitative measurement of EBOV sGP in animals, serum collected on pre-determined study days 0, 3, 4, 5, 6, at scheduled euthanasia or at moribund euthanasia, was analyzed using the EBOV Soluble GP (sGP) ELISA Kit (IBT Bioservices, Rockville, MD, USA); following manufacturer instructions. The reference standard provided in the kit was diluted to represent a range of 1.56 to 100 ng/mL, in order to achieve a more linear standard curve. Samples were tested at dilutions of 1:50 or 1:500, depending on the time point tested. The initial 1:50 dilution was prepared in manufacturer recommended buffer and subsequent ten-fold dilution (to achieve a final dilution of 1:500) was prepared in recommended buffer supplemented with 2% rhesus serum (BioIVT, Westbury, NY, USA) in order to maintain a consistent serum concentration across all dilutions. A quality control sample of sGP prepared in rhesus serum at a known concentration was also included to evaluate data quality and consistency.

### 2.13. Necropsy and Pathology

Following euthanasia, or after animals were found dead in the cage, a complete necropsy was conducted on each animal in the ABSL-4 laboratory. An examination and recording of findings of the external surfaces of the body, all orifices, and the cranial, thoracic and abdominal cavities and their contents were conducted. The following tissues were collected from each animal for viral load (plaque assays and qRT-PCR) and histopathology: exposure site (skin and underlying subcutis and muscle), right inguinal lymph node, axillary lymph node from the inoculated arm, mediastinal/hilar lymph node, mesenteric lymph node, right upper lobe lung (inflated), lower left lobe lung (uninflated), heart, spleen, liver, adrenal gland, kidney, stomach, duodenum, jejunum, ileum with gut associated lymphoid tissue (GALT), colon, rectum, brain, and any other gross lesion if any. Macroscopic necropsy observations were recorded on individual animal necropsy forms, to document location(s), size, shape, color, consistency, and number of lesions. Skin lesions were recorded on a petechia chart noting the location and description. Samples of tissues were aseptically removed and divided for viral load determination or fixed by immersion containers of 10% neutral-buffered formalin for a minimum of 14 days for histopathologic examination. Following fixation, tissue samples were embedded in paraffin, sectioned and stained with hematoxylin and eosin. Tissue sections were evaluated microscopically by a board-certified veterinary pathologist.

### 2.14. Quality Standards to Ensure Adequate and Well Controlled Study

All portions of this study performed at Texas Biomed adhered to a thorough Study Protocol, a quality agreement between Battelle Biomedical Research Center and Texas Biomed that assigned roles and responsibilities of study staff and was consistent with GLP principals, applicable Texas Biomed Standard Operating Procedures (SOPs), and generally recognized good documentation practices. All animal procedures were approved by the Institutional Animal Care Use Committee (IACUC). A Study Director was assigned as the primary point of control, and was responsible for the conduct of work and reporting of data generated at the facility. If any change to the approved protocol was required, an agreement to make the change was made in the form of a study protocol amendment and deviations from SOPs and were immediately reported to the Study Director and appropriately documented. All raw data and records required to reconstruct the study are maintained at Texas Biomed as specified in relevant Texas Biomed SOPs.

### 2.15. Blinding

The Veterinary Pathologist remained blinded to the group assignments during microscopic evaluation of stained slides. The pathologist was unblinded for preparation of the pathology report.

### 2.16. Statistics

Statistical analysis was performed at scheduled timepoints and for the combined terminal measurements for animals that were euthanized, if at least 2 animals per group were available for analysis. Preliminary analysis was performed to assess the model assumption of normality, to identify potential outliers, and to determine whether there are significant differences between the groups at baseline. For body weight, rectal body temperature, clinical chemistry, hematology, coagulation, biomarker, and sGP endpoints, ANOVA models were fitted to the change from baseline data separately at each time point to determine if there were significant changes from the pre-challenge baseline within a group, or differences between the groups. ANOVA models were used for convenience as there were only 2 groups in this study, the ANOVA models are equivalent to t-tests. Statistical analyses were conducted using SAS^®^ (version 9.4; SAS, Cary, NC, USA) on the 64-bit platform. Results are reported at the 0.05 level of significance.

## 3. Results

### 3.1. Survival

Twenty animals were inoculated, via intramuscular route, with 0.5 mL from a dilution of the starting EBOV viral stock corresponding to a dose of 1000 PFU. Dose delivery was confirmed through back titration (iteration 1, 665 PFU; iteration 2, 313 PFU). The four mock exposure controls (inoculated at ABSL-2 with 0.5 mL sterile saline) survived to scheduled euthanasia on day 6 post-exposure. The sixteen EBOV exposed animals assigned to serial euthanasia survived to euthanasia on days 3, 4, 5, or 6 post-exposure. Of the four EBOV exposed animals not scheduled for euthanasia: two succumbed to virus exposure on day 7 post-exposure (one euthanized and one found dead in cage (FDIC)), one animal was euthanized on day 9 post-exposure, and the final animal was FDIC on day 10 post-exposure (Table 2).

### 3.2. Clinical Signs of Infection (Clinical Scores, Body Temperature, and Body Weight)

**Figure 1:**
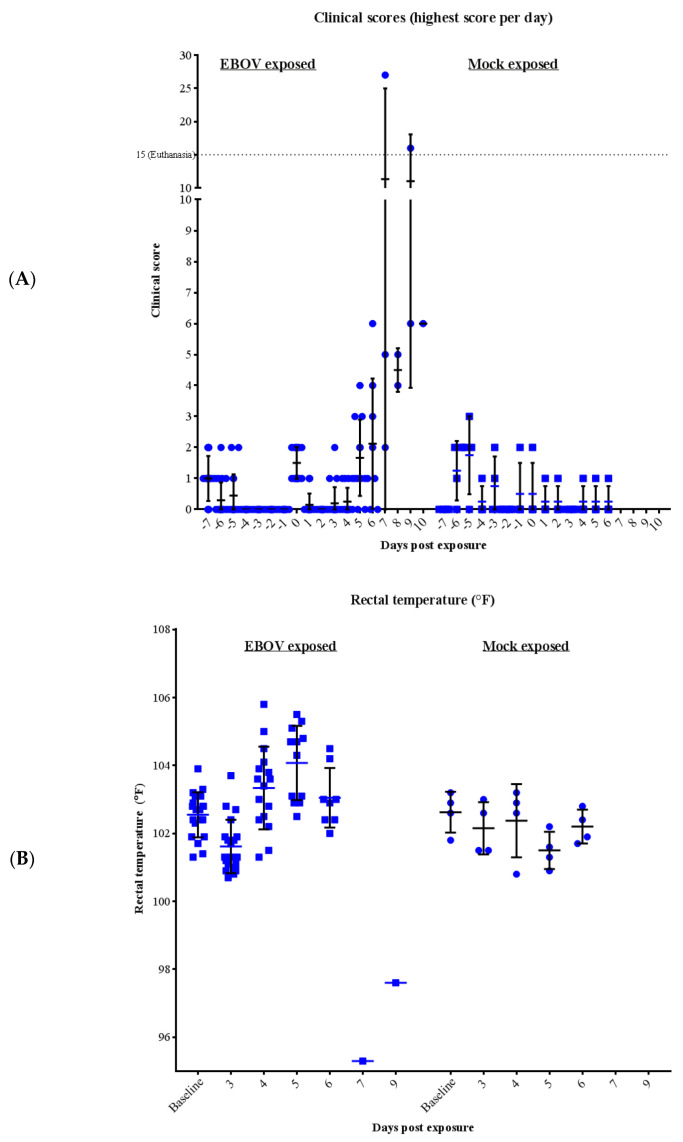
Daily clinical scores and rectal body temperature of rhesus macaques intramuscularly exposed with Ebola virus (EBOV) or mock exposed with phosphate buffered saline solution (PBS). Bars represent mean and standard deviation. (**A**) Maximum Daily Clinical Scores for each animal on each day are displayed. Euthanasia threshold is 15 (discontinuous line). (**B**) Febrile illness progression as demonstrated by rectal temperature over course of the study; for clinical scoring, baseline was defined as the average of rectal temperature on pre-exposure day −7 and day 0, exposure day; change from day 0 rectal temperature was significantly different between EBOV exposed and mock exposed control animals on day 5 (*p* = 0.0076) and day 6 (*p* = 0.0498).

Clinical scores are summarized in Figure 1 and the clinical scoring system is detailed in Table 1. All control animals at ABSL-2 remained healthy throughout the study and were euthanized as planned on day 6 post-exposure. During the majority of observations, mock exposure control animals did not exhibit any clinical signs other than unspecific signs of reduced feed consumption with a subsequent reduction of stool output. At time of scheduled euthanasia, all control animals had a clinical score of 0 or 1 and appeared clinically healthy.

Animals that were EBOV exposed did not exhibit disease specific clinical signs prior to day 3 post-exposure. During the early stages of the study, clinical scores above 0 were infrequent and similar to those of the control animals: reduced feed or fluid consumption with reduced stool output. The majority of animals scheduled for euthanasia on days 3 to 5 post-exposure had final clinical scores of 0, with only animal 086 and animal 026 having signs of mild petechial rash, increased rectal temperature (>2.8 °F, relative to baseline temperature), and/or reduced fluid intake. Abnormal responsiveness (diminished general activity or withdrawn) was observed on day 6 post-exposure. However, animals scheduled for euthanasia on day 6 appeared clinically healthy, with exception of animal 035 (presented with mild petechial rash on face and upper body). The severity of symptoms progressed between Study day 6 and day 10, as animals continued exhibiting reduced responsiveness along with signs of reduced feed and fluid intake, and reduced stool output. Animal 025 also presented with signs of bleeding in the stool and vomiting. During this time, the remaining four animals succumbed.

Changes in temperature were determined by comparing the baseline (average of each animal’s rectal temperature from the day they were transferred to ABSL-4 (day −7) and day 0, prior to EBOV exposure) with the individual rectal temperature taken at each scheduled time-point (Figure 1). The mock exposed animals did not exhibit temperature increases greater than 1 °F throughout the course of the study (relative to baseline). For EBOV exposed animals, on day 3 no animals exhibited increases greater than 1.1 °F. On days 4 to 5, animals began exhibiting rectal temperature increases greater than 2 °F (range of. 2.2 °F to 4 °F increases). On day 6, the final day of scheduled euthanasia, no animals exhibited an increase of more than 1.8 °F. Temperature decreases warranting a clinical score were less common until later in the study: on day 3, two animals exhibited temperature decreases of 2.0 to 2.9 °F and on day 5, one animal exhibited a decrease of 2.3 °F. On the day of death, two animals exhibited hypothermia; animal 029 (day 7) exhibited a 7 °F decrease in temperature (final recording was 95.3 °F) and animal 025 (day 9) exhibited a 4.8 °F decrease in temperature (final recording was 97.6 °F). The two other animals were FDIC, therefore body temperatures were not measured on the day of death.

The mean rectal temperature at day 0 was not significantly different between the groups (*p* = 0.5921) which implies that any differing group mean changes from day 0 were associated with the effects after exposure and not with inherent differences between the groups at baseline. Rectal temperature data model were fitted to untransformed values and no potential outliers were identified. Change from day 0 was not significant for mock exposed animals at any time point (*p* ≥ 0.05). For EBOV exposed animals, beginning on day 4, the mean increase from day 0 rectal temperature was statistically significant for EBOV exposed animals (*p* < 0.05). Mean increase from day 0 remained statistically significant (*p* < 0.05) on days 5 and 6, and change from baseline rectal temperature was significantly different between EBOV exposed and mock exposed control animals (*p* = 0.0076 and 0.0498, respectively).

Telemetry data showed that for EBOV exposed animals, the average of post exposure body temperatures was significantly greater than the average of baseline body temperatures (*p* < 0.05) for the majority of hourly average readings from days 3 through 9 post exposure. The shift from baseline body temperature for EBOV exposed animals was significantly greater than the shift from baseline for mock exposed control animals (*p* < 0.05) for the majority of hourly average readings from days 1 to 2, and days 4 through 6 post exposure. The shift from baseline activity for EBOV exposed animals was significantly less from the shift from baseline for mock exposed control animals (*p* < 0.05) for the majority of hourly average readings from days 3 through 6 post exposure.

Body weight changes were generally unremarkable throughout the study (data not shown). EBOV exposed animal 085 exhibited the largest decrease from baseline: decreases of 8.14% (day 4), 6.90% (day 5), and 7.31% (scheduled euthanasia, day 6). On Study day 3, the mean increase from baseline weight was statistically significant for animals in the EBOV exposed group (*p* < 0.05). There was no significant group effect on any study day (*p* ≥ 0.05).

### 3.3. Clinical Pathology: Hematology Analysis

Changes were observed in hematology, clinical chemistry, and coagulation parameters at pre-determined timepoints during the study and prior to euthanasia.

**Figure 2:**
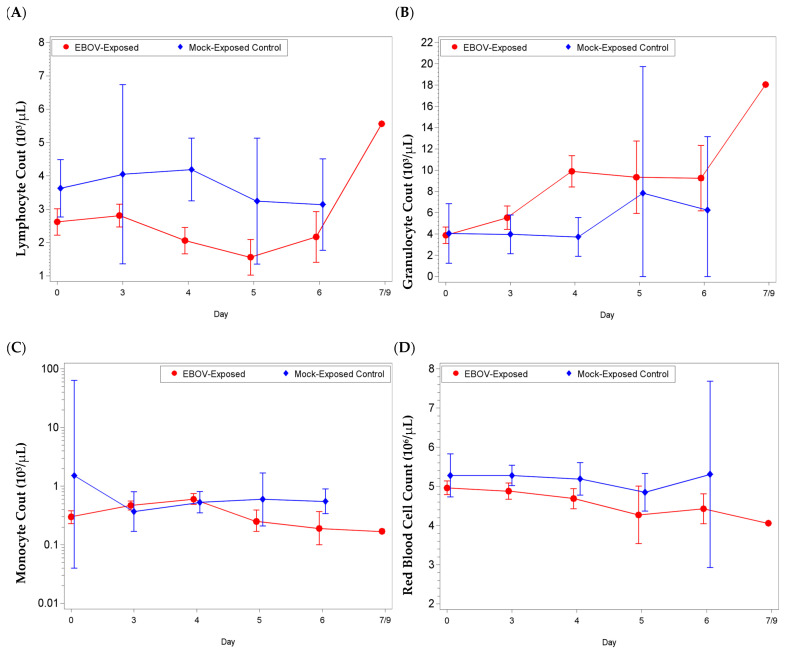
Cell Blood Counts in rhesus macaques intramuscularly exposed to EBOV or mock exposed with PBS. Blood was collected at pre-determined timepoints and prior to any euthanasia to determine the presence of changes in blood cells that correlate with disease. Group arithmetic means (lymphocytes and granulocytes) or geometric means (monocytes and red blood cells) with 95 percent confidence intervals over the course of the study (confidence intervals not plotted for sample sizes of less than 3 animals). Mean shifts from baseline (lymphocytes, granulocytes, and red blood cells) or shifts as a proportion of baseline (monocytes) were statistically compared between groups. (**A**) Lymphocyte counts; significant group effect on day 4 (*p* = 0.0031), (**B**) granulocyte counts; significant group effect on day 4 (*p* = 0.0006), (**C**) monocyte counts; significant group effects on days 3 and 4 (*p* < 0.05), and (**D**) red blood cell counts; significant group effects on day 4 (*p* = 0.0487).

Most noticeable changes were observed in lymphocyte (absolute and percentage) and granulocyte (absolute and percentage) values (Figure 2, Figure S1). For lymphocyte parameters, there were significant decreases from baseline for the EBOV exposed group on Study days 4, 5, and 6 (and day 3 for lymphocyte percentage; *p* < 0.05). The group effect for mean shift from baseline between EBOV exposed and mock exposed animals was significant on day 4 (absolute count *p* = 0.0031, percentage *p* = 0.0003). For granulocyte parameters, there were significant increases as a proportion of baseline for the EBOV exposed group on Study days 3, 4, 5, and 6 (*p* < 0.05) and the group effect was significant on day 4 (absolute count *p* = 0.0006, percentage *p* = 0.0004).

Concomitantly, monocyte values (reference range: 0.04–0.72 × 10^3^/µL, 0–2%) were elevated from reference range at various time points during the study; for absolute count, there were significant increases as a proportion of baseline for the EBOV exposed group on days 3 and 4, and a significant decrease as a proportion of baseline for the mock exposed group on day 3 (*p* < 0.05). The group effect for mean shift as a proportion of baseline between EBOV exposed and mock exposed animals was significant on days 3 and 4 (*p* < 0.05).

Red blood cell values (RBC, reference range: 4.00–6.6 × 10^6^/µL), for EBOV exposed animals were rarely below reference range. However, animals 033, 083, and 029, had RBC counts below reference range on the day of euthanasia (study day 5, day 6 and day 7, respectively) and there were significant decreases from baseline for the EBOV exposed group on days 4 and 5 (*p* < 0.05). Only minor changes were observed for mock exposed animals. The group effect for mean shift from baseline between EBOV exposed and mock exposed animals was significant on day 4 (*p* = 0.0487).

### 3.4. Clinical Pathology: Clinical Chemistry Analysis

**Figure 3:**
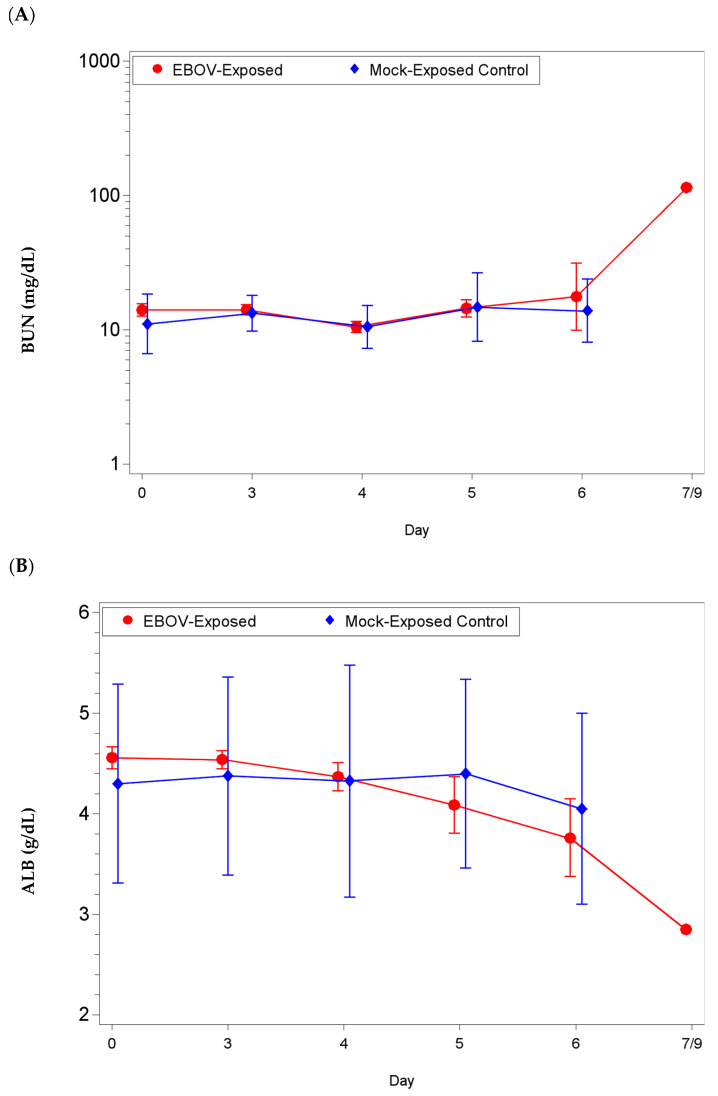
Clinical Chemistry Parameters in rhesus macaques intramuscularly exposed to EBOV or mock exposed with PBS. Blood was collected at pre-determined timepoints and prior to any euthanasia to determine the presence of biochemical markers that correlate with clinical pathology (hepatic and/or renal function). Group geometric (BUN) or arithmetic (ALB) means with 95 percent confidence intervals over the course of the study (confidence intervals not plotted for sample sizes of less than 3 animals). Mean shift as a proportion of baseline (BUN) or mean shift from baseline (ALB) were statistically compared between groups. (**A**) Blood urea nitrogen, significant group effect on day 3 (*p* < 0.05), (**B**) albumin, significant group effect on day 5 (*p* < 0.05).

Several EBOV exposed animals showed increases in liver transaminase values indicative of hepatic injury, as well as abnormal kidney parameters (Figure S2 and Figure 3). The majority of EBOV exposed animals euthanized on, or after, day 5 post-exposure exhibited alanine aminotransferase (ALT, reference range: 5–61 U/L), gamma-glutamyl transferase (GGT, reference range: 22–53 U/L) and alkaline phosphatase (ALP, reference range: 45–661 U/L) values higher than baseline on day of euthanasia, in the latter stages of the disease. For ALP, there were no significant changes as a proportion of baseline for either group (*p* ≥ 0.05) but the group effect for mean shift as a proportion of baseline was significant on day 4 (*p* < 0.05; Figure S2). For ALT, there were significant increases as a proportion of baseline for the EBOV exposed group on days 5 and 6 (*p* < 0.05); there was no significant group effect on any study day (*p* ≥ 0.05; Figure S2). For GGT, there were significant decreases as a proportion of baseline for the EBOV exposed group on days 3 and 4 and a significant increase as a proportion of baseline for the EBOV exposed group on day 6 (*p* < 0.05); there was no significant group effect on any study day (*p* ≥ 0.05). Animals exhibited total bilirubin (TBIL) values within the reference range (0.1–1.2 mg/dL) throughout the study (data not shown); on day 4, there were significant decreases as a proportion of baseline for the EBOV exposed and mock exposed groups, and the group effect for mean shift as a proportion of baseline was significant on day 6 (*p* < 0.05). Mock exposed animals did not exhibit ALT, ALP, GGT, and/or TBIL changes during the course of the study (*p* ≥ 0.05).

Evidence of moderate to marked impaired renal function (up to 3-fold increases in blood urea nitrogen (BUN; reference range: 8–38 mg/dL) and decreased albumin (ALB; reference range: 3.6–5.5 g/dL)) was also observed in EBOV exposed NHPs immediately prior to euthanasia (Figure 3). Mock exposed animals exhibited BUN and ALB values within the reference range throughout the study. For BUN: there was a significant decrease as a proportion of baseline for the EBOV exposed group on day 4 (*p* < 0.05), a significant increase as a proportion of baseline for the mock exposed group on days 3 and 5 (*p* < 0.05), and a significant group effect for mean shift as a proportion of baseline on day 3 (*p* < 0.05). For ALB: there were significant decreases from baseline for the EBOV exposed group on days 5 and 6 (*p* < 0.05), and a significant group effect on day 5 (*p* < 0.05), concomitant with increased hepatic enzymes. 

### 3.5. Clinical Pathology: Coagulation Analysis

**Figure 4:**
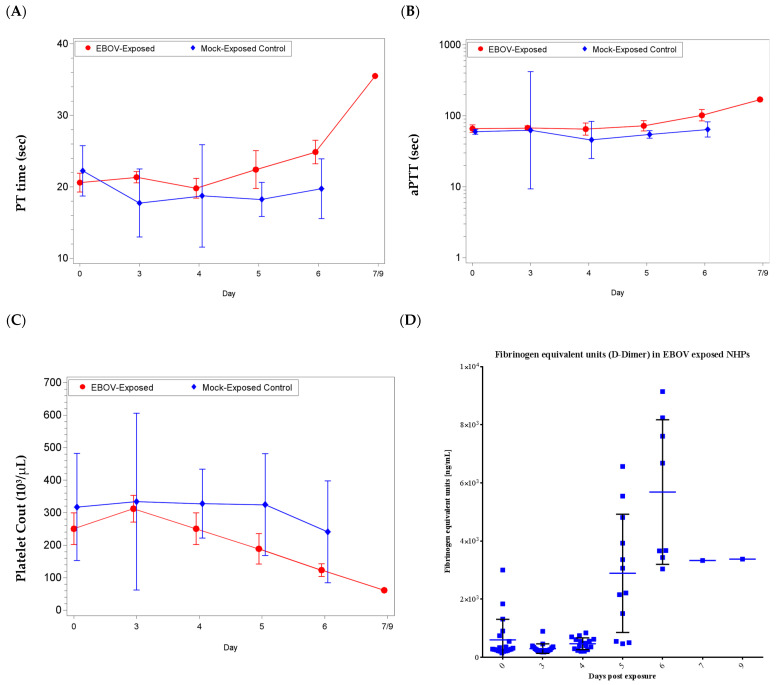
Coagulation parameters in rhesus macaques intramuscularly exposed to EBOV or mock exposed with PBS. Group arithmetic (prothrombin (PT) time and platelet counts) or geometric (activated partial thromboplastin (aPTT) and D-Dimers) means with 95 percent confidence intervals over the course of the study (confidence intervals not plotted for sample sizes of less than 3 animals). Mean shifts from baseline (PT time and platelet counts) or shifts as a proportion of baseline (aPTT) were statistically compared between groups. (**A**) Clotting times: PT time; significant group effects on days 3, 5, and 6 (*p* < 0.05), (**B**) clotting times: aPTT; no significant group effects (*p* ≥ 0.05), (**C**) platelet counts; no significant group effects (*p* ≥ 0.05), and (**D**) presence of D-Dimers (no data for mock exposed animals).

Coagulopathy was present in some EBOV exposed animals, evidenced by elevated coagulation times (activated partial thromboplastin (aPTT) and prothrombin (PT) time) in the latter stages of infection, as early as day 5 up to day 9 post-exposure (Figure 4).

For PT time, there was a significant increase from baseline for the EBOV exposed group on day 6 and a significant decrease from baseline for the mock exposed group on day 3 (*p* < 0.05); the group effect for shift from baseline was significant on days 3, 5, and 6 (*p* < 0.05). For aPTT, there was a significant increase as a proportion of baseline for the EBOV exposed group on day 6 (*p* < 0.05); there were no significant group effects (*p* ≥ 0.05).

In addition to coagulopathy, reduced platelet counts (reference range: 230–650 × 10^3^/µL), and an increase in D-dimers were observed. Twelve EBOV exposed animals exhibited low platelet counts on the day of euthanasia or the sampling timepoint immediately prior to the animals being FDIC (as early as day 4 and up to day 6). There was a significant increase from baseline for the EBOV exposed group on day 3 and significant decreases from baseline for the EBOV exposed group on days 5 and 6 (*p* < 0.05); there were no significant group effects (*p* ≥ 0.05).

Elevated D-Dimer is often observed in cases of disseminated intravascular coagulation [27,28]. Consistent with overall animal health and the individual animal coagulation data, increases for D-Dimers were not observed on day 3 or day 4 post-exposure. However, beginning on day 5 post-exposure and similar to coagulation data, D-Dimers were increased on the day of euthanasia for animals euthanized after day 4 (with the exception of animal 080; Figure 4). There was a significant increase as a proportion of baseline for the EBOV exposed group on days 5 and 6 (*p* < 0.05). Increased coagulation parameters and D-dimer values were considered consistent with dysfunction in coagulation, which is a feature of EVD in humans.

### 3.6. Viral Load

Levels of infectious EBOV in the blood as determined by plaque assay were evaluated (Table 3). The presence of viral genomic RNA was evaluated by qRT-PCR (targeting a region of glycoprotein) using serum samples (Table S1) from scheduled timepoints. Viral load in tissues taken at scheduled or moribund euthanasia was also evaluated via plaque assay and qRT-PCR (Tables S2 and S3, Table 4 and Table 5).

Low levels of virus were detected as early as day 3 with geometric mean values of 9.55 × 10^−01^ PFU/mL (95% confidence interval: 3.62 × 10^−01^, 2.52 × 10^0^) by plaque assay and 4.43 × 10^0^ (95% confidence interval: 7.19 × 10^−01^, 2.73 × 10^1^) genome equivalents (GE)/mL by qRT-PCR. By day 4 post exposure, 69% (11 of 16) of EBOV exposed animals exhibited detectable levels of infectious virus in the serum, ranging from 2.25 × 10^1^ to 2.04 × 10^6^ PFU/mL (2.14 × 10^3^ to 1.17 × 10^5^ GE/mL). After day 4 post exposure, all animals exhibited detectable levels of infectious virus in the serum until death. Peak viremia was observed between day 5 and day 6 at a concentration that ranged between 1.53 × 10^2^ and 2.36 × 10^7^ PFU/mL (2.32 × 10^4^ to 6.08 × 10^8^ GE/mL). Animal 029 (succumbed on day 7) had titers of 3.70 × 10^6^ PFU/mL by plaque assay (day 7) and 3.94 × 10^8^ GE/mL (day 6).

Viral load in tissues (measured by plaque assay and qRT-PCR) was similar to viremia observed in serum collected at time of euthanasia. Infectious EBOV was detected as early as day 3 post-exposure in tissues from the exposure site (skin and underlying subcutis and muscle), axillary lymph node (from the inoculated arm) and right inguinal lymph nodes, spleen, liver, lung, small (ileum) and large (colon and rectum) intestines (Tables S2 and S3, Table 4 and Table 5). Genomic RNA was detected as early as day 3 post-exposure in all tissues except lung, stomach, duodenum and ileum.

More than half of EBOV exposed animals exhibited positive viral load by plaque assay on all tissues, except for brain, stomach and duodenum by plaque assay. All animals with scheduled euthanasia on day 4 and day 5 exhibited detectable levels of infectious virus in the majority of the evaluated tissues. Animals euthanized on or after day 6 post-exposure had detectable infectious virus in all tissues tested with the exception of animal 024 (levels were undetectable in the brain and kidney). Infectious virus was most frequently detected in spleen (19 of 20 animals), lymph nodes (17 of 20), and liver (16 of 20). Genomic RNA was frequently detected in all tissues except the lung and parts of the gastrointestinal tract.

### 3.7. Cytokines and Chemokines Analysis

**Figure 5:**
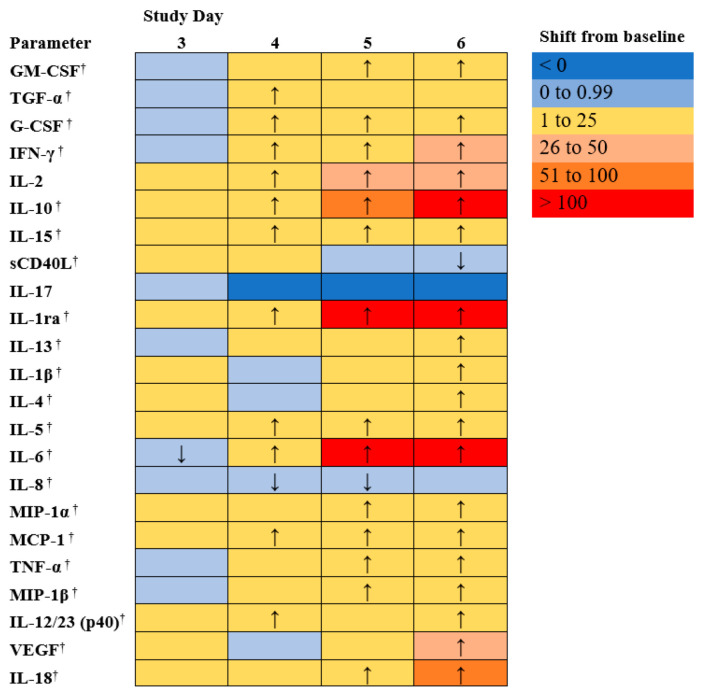
Cytokines and chemokines. Mean shift as a proportion of baseline for cytokines and chemokines in rhesus macaques intramuscularly exposed with EBOV Kikwit variant; ↑ indicates that the mean on the study day was significantly greater than that at baseline (day 0) (*p* < 0.05); ↓ indicates that the mean at the study day was significantly less than that at baseline (day 0) (*p* < 0.05); † indicates that values for this parameter were log-transformed for the analysis.

Immunological response to EBOV exposure was assessed during the course of this study. EBOV induces high levels of pro-inflammatory cytokines and chemokines [29,30,31]. Cytokine and chemokine profiles were analyzed using serum from individual animals, collected on days 0, 3, 4, 5, 6, and at euthanasia. Figure 5 shows the mean shift as a proportion of baseline for each of 23 cytokine or chemokines measured.

Humans infected with EBOV have shown high levels of pro-inflammatory cytokines (e.g., IL-1β, IL-4, IL-1RA, IL-6, IL-8, IL-15 and IL-16) and chemokines (e.g., MIP-1α, MIP-1β, MCP-1) [29,30,31]. In addition, high levels of sCD40L have been detected in survivors leading to the suggestion that sCD40L could be a novel biomarker to predict clinical outcome. One hypothesis is that the elevated levels of sCD40L detected in survivors could be a result of ongoing repair of altered endothelium by activated platelets [32,33]. There were significant increases as a proportion of baseline for the EBOV exposed animals on day 6 for sCD40L (*p* < 0.05).

On average, in the NHPs in this study, increased levels of IL-1RA, IL-8, and MCP-1 were detected, similar to what has been reported in humans infected with EBOV [34,35]. Further, decreased levels of sCD40L were found, which is expected in non-survivors based on the human data [36].

Many interleukins which may be involved in inflammation, coagulopathy and endothelial permeability during EVD (5) were increased, generally after day 4 post exposure. For EBOV exposed animals, the following parameters exhibited significant (*p* < 0.05) early changes from baseline: TGF-α, G-CSF, IFN-γ, IL-2, IL-10, IL-15, IL-1ra, IL-5, IL-6, MCP-1, and IL-12/23 (p40) exhibited significant increase from baseline on Study day 4. IL-18 (a proinflammatory cytokine involved in induction of interferon gamma; significantly increased on days 5 and 6) and IL-13 exhibited some increases (significantly increased on day 6 post-exposure), but overall exhibited fewer changes than the other interleukins. Changes in IL-17 (a pro-inflammatory cytokine) were sporadic and did not follow a clear pattern (differences from baseline were not significant, *p* ≥ 0.05). Interferon γ increased in most animals after day 3 post exposure (significant increase from baseline on days 4, 5, and 6, *p* < 0.05); IFN α and β were not measured in this panel, but in EVD, Type 1 Interferon responses have been observed to be impaired, which can lead to increased Type II interferon (5). VEGF, G-CSF, and GM-CSF, which are involved in growth differentiation, were significantly increased (*p* < 0.05). MCP-1, MIP-1α, and MIP-1β, involved in attracting immune cells to the site of inflammation and may be involved in immunopathology during EVD (5), were significantly increased as well on days 4 or 5 and through day 6 (*p* < 0.05). In addition, there were significant increases as a proportion of baseline for the EBOV exposed animals on days 5 and 6 for TNF-α, and on day 6 for IL1 β and IL-4 (*p* < 0.05).

As with other parameters discussed above, changes were more frequent and more drastic in animals euthanized later during the study.

**Figure 6:**
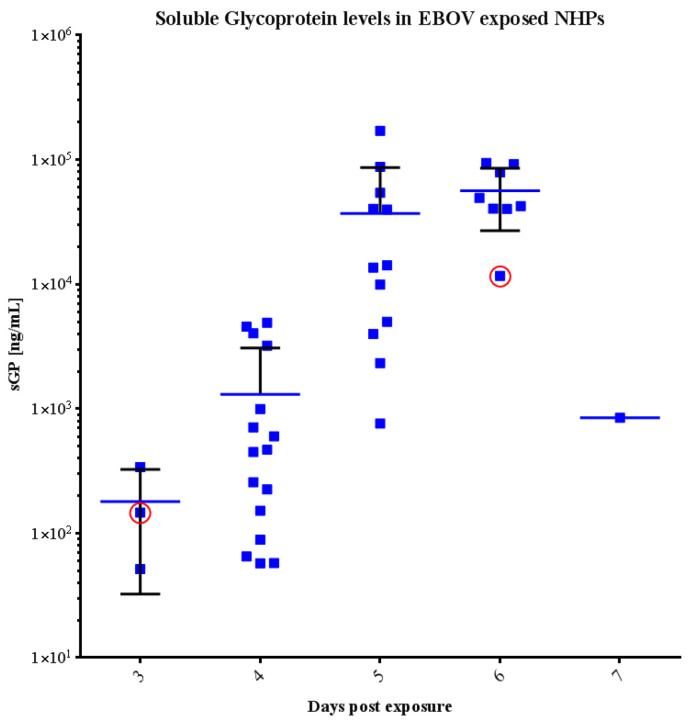
Soluble glycoprotein (sGP) levels in nonhuman primate (NHP) serum or plasma after EBOV exposure. Bars represent mean and standard deviation. The following samples used plasma instead of serum for analysis: day 3 for animals 025, 080, 087, (below limit of detection, not represented on graph) and 029 (circled in red); day 6 for animal 025 (circled in red).

The amount of soluble glycoprotein (sGP) in serum collected on pre-determined days 0, 3, 4, 5, 6 and at time of euthanasia was determined using the EBOV Soluble GP ELISA Kit (IBT Bioservices), which allows for quantitative measurement of EBOV sGP in non-human primate serum (Figure 6). Overall, sGP was first detected in three animals on day 3 post-exposure (geometric mean: 1.37 × 10^2^ ng/mL; 95% confidence interval: 1.31 × 10^1^, 1.44 × 10^3^ ng/mL) and all animals on day 4 post-exposure (geometric mean: 4.63 × 10^2^ ng/mL; 95% confidence interval: 1.99 × 10^2^, 1.07 × 10^3^ ng/mL). Levels increased over Study days 3 through 6 and dropped at time of euthanasia.

### 3.8. Gross Necropsy

**Figure 7:**
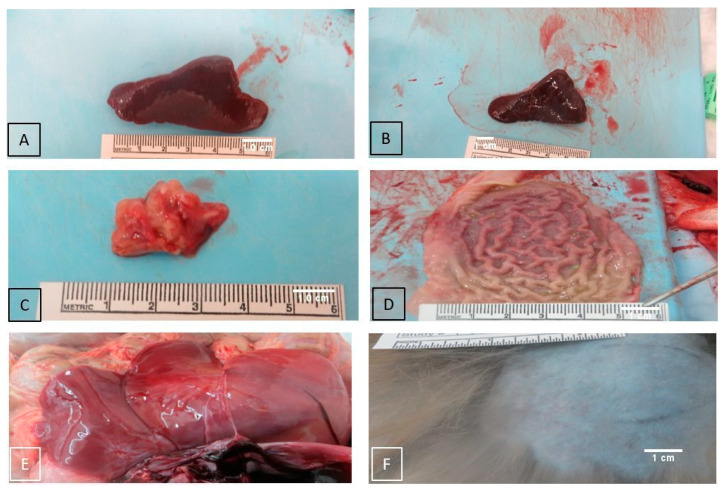
Gross necropsy. Gross findings at necropsy in rhesus macaques intramuscularly exposed to EBOV Kikwit. The most common findings were: (**A**) animal 026 (scheduled euthanasia, day 5) rounded, enlarged, and firm spleen; (**B**) animal 081 (scheduled euthanasia, day 3, normal spleen); (**C**) animal 083 (scheduled euthanasia, day 6) enlarged axillary lymph node; (**D**) animal 030 (scheduled euthanasia, day 4), red mucosa in gastrointestinal tract (stomach); (**E**) animal 024 (scheduled euthanasia, day 6) pale liver; (**F**) animal 035 (scheduled euthanasia, day 6) petechial skin rash, exposure site.

Table S4 summarizes the gross findings at necropsy for EBOV exposed animals and Figure 7 shows representative images of common findings. Mock exposed animals were normal at gross examination and no significant lesions were found; the following minor observations were made: animal 089 had two ulcers (2 × 3 cm^2^) in the proximal colon and animal 084 had a focal (1 × 1 cm^2^) area of subcutaneous hemorrhage on the skull in the periorbital area.

Animals exposed to EBOV presented with macroscopic findings consistent with EBOV exposure in NHPs (percentages based on EBOV exposed animals, *n* = 20). Rash at the site of exposure was observed in 15% (*n* = 3) and 45% (*n* = 9) had a petechial rash somewhere on the body. Lymph node abnormalities (enlargement and/or discoloration and/or firmness) were common: axillary, 95% (*n* = 19); mediastinal, 40% (*n* = 8); mesenteric, 25% (*n* = 5); and inguinal, 75% (*n* = 15). Spleen abnormalities (enlargement and/or rounded and/or firmness) were observed in 60% (*n* = 12), and liver abnormalities (pale/discoloration and/or friability) were observed in 35% (*n* = 7). Gastrointestinal tract findings included: 35% (*n* = 7) exhibited red mucosa in the stomach, duodenum, or colon and 35% (n = 7) had blood present in the lumen of the rectum. Some other findings occurred in fewer than 5 animals: 20% (*n* = 2 of 10 females) exhibited blood in the lumen of the uterus; 20% (*n* = 2 of 10 males) exhibited blood in the testes; 15% (*n* = 3) exhibited petechia or red mucosa in the urinary bladder; 10% (*n* = 2) had enlarged adrenal gland(s); and 10% (*n* = 2) had red kidneys. Other findings were only observed in a single animal. Animal 079 had a firm adrenal gland and in animal 026 the underlying subcutis was wet at the site of exposure. Animal 023 had reddened skin at the exposure site, dark kidneys, and a firm ileum. Animal 025 had red mucosa in the jejunum, and dark red mucosa in the ileum and rectum. Animal 029 had blood in the ventricles of the brain; at the time of euthanasia; this animal had mild petechia in the axillary and inguinal areas, and on the face, but this was not observed during necropsy and is thus not noted in the gross pathology table.

The earliest and most consistent EBOV related macroscopic observation was dark and/or firm inguinal and axillary lymph nodes noted at day 3 post-exposure. Firm, rounded spleen was first observed at day 4 post-exposure. Petechial rash, pale liver, urinary bladder mucosal petechia and changes at the challenge site were first noted at day 5 post-exposure. The full spectrum of EBOV-related observations were noted in animals euthanized or found dead between days 6 to 10 post-exposure.

### 3.9. Histopathology

**Figure 8:**
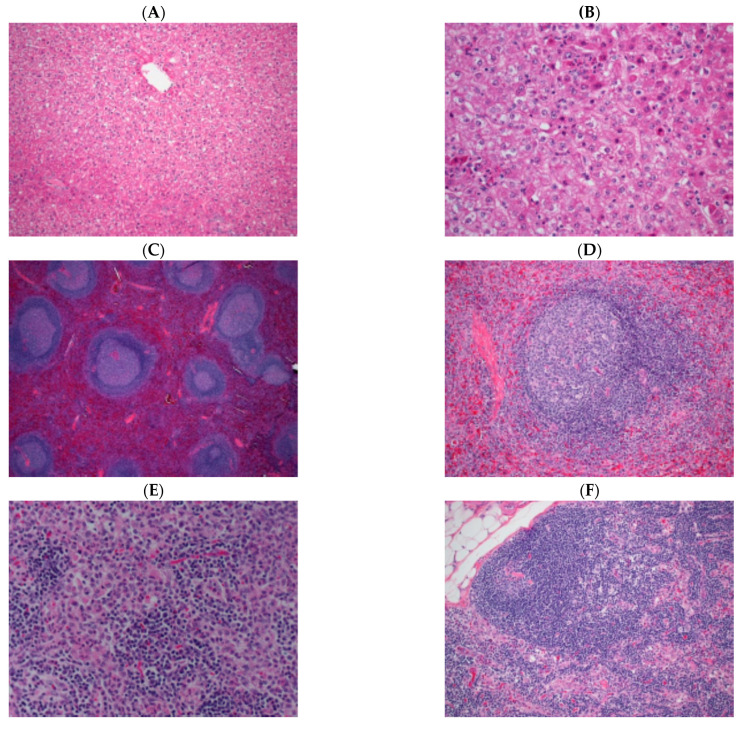
Histopathological representative images. (**A**) Liver, animal 030 (scheduled euthanasia, day 4). Essentially normal tissue; 20×. (**B**) Liver, animal 033 (scheduled euthanasia, day 5). Hepatocellular necrosis with inflammation; 40×. (**C**) Spleen, animal 030 (scheduled euthanasia, day 4). Essentially normal tissue; 4×. (**D**) Spleen, animal 033 (scheduled euthanasia, day 5). Lymphoid depletion with lymphocytolysis. Note fibrin deposition surrounding follicle; 20×. (**E**) Axillary lymph node, animal 082 (scheduled euthanasia, day 4). Sinus histiocytosis and minimal acute inflammation; 20×. (**F**) Inguinal lymph node, animal 033 (scheduled euthanasia, day 5). Decreased cortical lymphocytes with reduced size and number of follicles, sinus histiocytosis and hemorrhage; 20×.

Representative images of common microscopic findings are shown in Figure 8. The earliest and most consistent microscopic finding at day 3 post-exposure was lymph node sinus histiocytosis (also noted in mock exposed animals euthanized on day 6); hemorrhage within the lymph node was noted in one animal and hemorrhage was noted in the rectum of two animals. The most consistent microscopic finding at day 4 was lymph node sinus histiocytosis but additional changes noted in the axillary lymph node consisted of decreased cortical lymphocytes, lymphocytolysis within germinal centers and minimal acute inflammation. Fibrin deposition in the spleen and lymph node and hepatocellular necrosis were noted first at day 5. The full spectrum of histopathologic changes typical of experimental EBOV exposure in macaques were noted in NHPs euthanized or found dead between days 7 to 10 post-exposure, including: inflammation at the challenge site; lymph node cortical lymphoid depletion, lymphocytolysis, sinus histiocytosis, inflammation, fibrinoid vasculitis, fibrin and hemorrhage; splenic lymphoid depletion, lymphocytolysis, fibrin and marginal sinus congestion/hemorrhage; hepatocellular single cell necrosis and inflammation; adrenal gland necrosis and inflammation; renal medullary thrombosis; and duodenal hemorrhage. The temporal progression of findings were similar to that described in rhesus macaques experimentally exposed to EBOV via the aerosol route [37].

Microscopic findings in mock exposed animals included lymphoid depletion and sinus histiocytosis but changes were of minimal to mild severity grade, and are findings commonly noted as spontaneous change in macaques. The primary differentiating feature when lymph node and spleen from mock exposed and EBOV exposed monkeys were compared was the absence of ancillary findings such as necrosis, hemorrhage and fibrin deposition in mock exposed animals and the magnitude of severity grade of lymphoid depletion in EBOV exposed animals.

### 3.10. Onset to Abnormality

For time from challenge to onset of abnormality, nine parameters were statistically significantly different (*p* < 0.05) between EBOV and mock exposed animals: GGT, granulocytes, lymphocytes, monocytes, rectal temperature, and telemetry body temperature. Because there were only four mock exposed control animals and only four animals were followed to moribund euthanasia or death, the statistical analysis was limited, and one should use caution when interpreting statistical analysis results.

Table 6 shows the average time of abnormality onset for select parameters measured within the study. The majority of parameters had an average onset of approximately 4 days; sCD40L decreases exhibited an early average onset of 3.50 days (range of 3 to 4 days), while BUN decreases exhibited a later average onset of 5.18 days (range of 3 to 9 days).

## 4. Discussion

The purpose of this study was to further characterize the disease course in rhesus macaques exposed intramuscularly to a target dose of 1000 PFU *Zaire ebolavirus* (Ebola virus (EBOV), Kikwit variant) or PBS. These data support the reproducibility of the rhesus mode of EVD and expand upon previous descriptions. Twenty animals were housed at ABSL-4 for exposure to EBOV and four animals were housed at ABSL-2 as mock exposed controls. Changes in body weight, rectal temperature, clinical pathology, and viremia were evaluated throughout the study. Sixteen animals were euthanized on schedule in order to characterize pathology from multiple timepoints post-exposure. To confirm that the exposure dose was lethal and to measure physiology and clinical pathology, four EBOV exposed animals were not scheduled for euthanasia; two of these animals succumbed to virus exposure on day 7 post-exposure (one euthanized and one was found dead in cage (FDIC)), one was euthanized on day 9 post-exposure, and the fourth animal was FDIC on day 10 post-exposure.

Complete blood counts and biochemical analyses were performed on whole blood collected from EBOV infected macaques at pre-determined time points during the study and prior to euthanasia. All animals had lymphocyte counts below the normal range for many of the sampling timepoints and most animals exhibited granulocytosis. Although lymphopenia has been associated with EBOV infection in humans, both lymphopenia and granulocytosis are changes that also may arise from stress, as observed in mock exposed animals [20,24,38]. The lymphopenia observed in the EBOV exposed animals may be associated with the bystander apoptosis of lymphocytes in EBOV infections. However, because abnormal values were observed for mock exposed animals, it is difficult to use this biomarker as an unambiguous sign of EBOV infection; though the difference in shift from baseline was significant between EBOV and mock exposed animals on day 4.

Clinical chemistry parameters that are frequently indicative of EVD in humans (indicative of marked liver malfunction; increases in ALT, ALP and GGT) were observed in some animals between days 5 to 9 post-exposure; significant differences when compared to baseline were observed in EBOV exposed animals for ALT and GGT. However, biochemical evidence of moderate to marked impaired renal function (increases in BUN and decreased ALB) was not generally observed in EBOV exposed animals; though there were significant decreases from baseline ALB for the EBOV exposed group on days 5 and 6. Clotting time was measured with two tests (aPTT and PT) at scheduled time points post-exposure and prior to euthanasia. When compared to pre-exposure values, several EBOV exposed animals had prolonged clotting times on or near the day of euthanasia. The EBOV exposed group experienced significant increases from baseline for both aPTT and PTT on day 6. For D-Dimer, the EBOV exposed group experienced significant increase from baseline on days 5 and 6.

There were few, if any indications of EBOV infection on day 3 post-exposure. Only sporadic evidence of infectious virus as measured by plaque assay or viral copies by qRT-PCR was observed. The only pathological evidence of infection was noted in the lymph nodes. By day 4 post-exposure a majority of the animals presented evidence of infection through detection of live virus or RNA copies by plaque assay and qRT-PCR, although the pathological evidence of infection was similar to day 3 post-exposure in that the most obvious changes were in the lymph nodes. By day 5 post-exposure, there was universal evidence for infection from plaque assay and qRT-PCR data and systemic macroscopic and microscopic findings of necrosis, inflammation, fibrin deposition and hemorrhage in multiple tissues. Animals euthanized after day 5 showed evidence for systemic infection both by the presence of virus and RNA in most tissues and widespread pathological evidence of infection.

**Figure 9:**
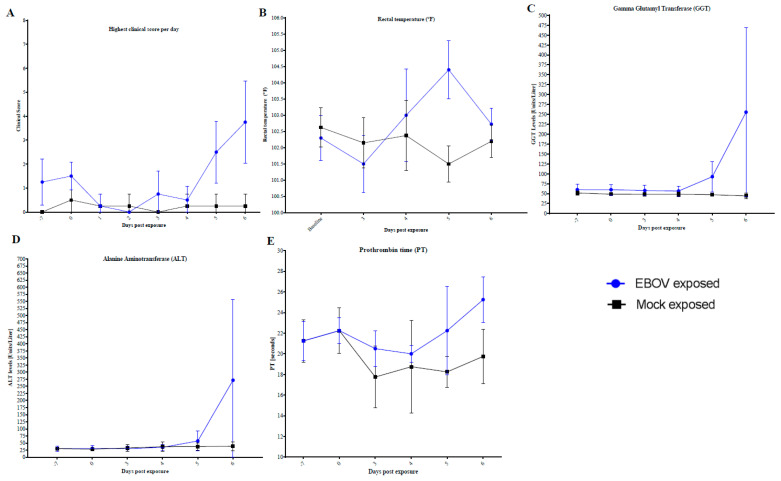
Biomarkers over time course in EBOV exposed versus mock exposed animals. Mean and standard deviation are shown. Four EBOV exposed animals are represented: animals not scheduled for serial euthanasia, in order to analyze changes in physiology and clinical pathology kinetics. (**A**) Highest clinical score per day. (**B**) Rectal Temperature. (**C**) Gamma Glutamyl Transferase. (**D**) Alanine Aminotransferase. (**E**) Prothrombin time.

In addition to the detection of infectious virus by plaque assay and RNA copies via qRT-PCR, there were other biomarkers that reliably correlated with EBOV-induced disease, based on comparing the data from the EBOV exposed to the mock exposed animals (Figure 9). These biomarkers include: clinical scores over 3, rectal temperature changes of more than 2.5 °F, GGT values above the reference range, marked elevations in ALT or ALP (greater than two-fold increase), and coagulopathy. During necropsy, animals exposed to EBOV presented with common macroscopic findings such as: lymph node abnormalities (changes in size, color, and consistency), spleen abnormalities (changes in size, color, and consistency), blood in the lumen of the rectum, petechial rash, or liver abnormalities (changes in color and consistency); none of which were ever detected in mock exposed animals. Other biomarkers, primarily because of their variable and intermittent high values before exposure, were not unambiguous indications of disease such as increased BUN, decreased ALB, and prolonged clotting times although these parameters did appear to be consistent at later stages of the disease (i.e., after day 6 post-exposure).

The data in this study supports the observation that EBOV exposure in rhesus macaques is a rapid systemic disease similar to the infection in humans under a compressed time scale [2,39,40,41]. The disease appears to begin as a local infection, most likely including the lymph nodes draining the site of injection. As late as day 3 post-exposure there is little evidence for circulating virus or systemic spread to other organs. The local infection observed on day 3 post-exposure transitions from a local to a systemic infection with high levels of virus in the blood and most organs in every animal by day 5 post-exposure. After the establishment of this systemic infection there is a short, intensive disease that results in euthanasia or death a few days later (median 8.29 days). Indicators of infection became evident when disease reached the systemic state at day 5 post-exposure at which time a number of biomarkers were evident of infection including viral load by plaque assay, RNA copy number by PCR, sGP, rectal temperature changes (greater than 2.5 °F), and liver enzyme elevation (e.g., GGT, ALT, ALP).

## 5. Conclusions

In summary, this study was designed—and supported by the Biomedical Advanced Research and Development Authority (BARDA)—to more completely characterize the disease course of EBOV in NHPs and to determine if the model could be reliable for the evaluation of medical countermeasures against EBOV. As such, this study was conducted following the intent of the Animal Rule regulations in order to ensure the study would meet FDA standards of being adequate and well controlled. All portions of this study adhered to a thorough and preapproved Study Protocol, a quality agreement between Battelle Biomedical Research Center and Texas Biomed, applicable Texas Biomed SOPs, and generally recognized good documentation practices.

Many of the parameters measured in this study have also been measured in previous studies [17,18,20,21], thus confirming the reproducibility of the model. The strategy of scheduled euthanasia permitted evaluation of infection at the earliest stages of the disease in order to determine if we can correlate the results from the measured biomarkers with symptoms and the pathological spread of the disease. The emphasis on the early stages of the disease also provides an opportunity to identify potential triggers that are appropriate for intervention in a therapeutic model.

This model was previously used to evaluate potential therapeutic countermeasures for BARDA, and two therapeutic antibody cocktails demonstrated a statistically significant increase in survival when used to treat EBOV infected NHPs at day 5 post-exposure [42]. These therapeutic countermeasures were then used in the PALM trial during the 2018–2019 EBOV in the Democratic Republic of the Congo [43] and subsequently underwent evaluation by the Center for Drugs Evaluation and Research (CDER) for approval, based on their demonstration of clinical benefit. We believe these results demonstrate that this model, when correctly used, is critical tool for identifying effective countermeasures against EBOV.

## Figures and Tables

**Table 1:** Clinical observation parameters and numerical scores.

	Numerical Score and Description					
**Parameter**	0	1	2	3	8 ^1^	15 ^2^
Feed	25–100% consumed	<25% consumed	N/A	N/A	N/A	N/A
Food Enrichment	25–100% consumed	<25% consumed	N/A	N/A	N/A	N/A
Dehydration	Normal	≥2 s TENT test	N/A	N/A	N/A	N/A
Hair Coat	Normal appearance	Rough hair coat	N/A	N/A	N/A	N/A
Nasal Discharge	None	Present	N/A	N/A	N/A	N/A
Body Weight	0% to <10% decrease	≥10% to <20% decrease	≥20% decrease	N/A	N/A	N/A
Fluid	Normal	Reduced intake	Not drinking	N/A	N/A	N/A
Stool	Normal	No stool	Diarrhea or reduced	N/A	N/A	N/A
Bleeding	None	At blood collection site	Other than collection site	N/A	N/A	N/A
Temperature	<2 °F change	2–2.9 °F change	3–4.9 °F change	≥5 °F ^1^	N/A	N/A
Petechia	None	Mild, 1–39% of body	Moderate, 40–79% of body	Severe, ≥80% of body	N/A	N/A
Respiration	Normal	N/A	N/A	N/A	Labored	Agonal ^2^
Responsiveness	Alert, responsive, normal activity	Slightly diminished activity; responds normally to stimuli	Withdrawn, reduced response to stimuli	N/A	Moderate to dramatically reduced response to stimuli ^1^	Severely/completely unresponsive ^2^

N/A—not applicable, number not used for this parameter; ^1^ Secondary euthanasia criteria, score of 8 for responsiveness and: greater than 5 degree temperature change OR two of certain clinical chemistry parameters above specified range; ^2^ Primary euthanasia criteria (score of 15 or greater).

**Table 2:** Scheduled euthanasia and mortality summary.

Animal ID	Sex	Exposure Group	Day of Death	Final Clinical Score
028 *	Male	EBOV	Scheduled, day 3	0
031 *	Female	EBOV	Scheduled, day 3	0
081 **	Female	EBOV	Scheduled, day 3	0
088 **	Male	EBOV	Scheduled, day 3	0
027 *	Male	EBOV	Scheduled, day 4	0
030 *	Female	EBOV	Scheduled, day 4	0
082 **	Female	EBOV	Scheduled, day 4	0
087 **	Male	EBOV	Scheduled, day 4	0
026 *	Male	EBOV	Scheduled, day 5	2
033 *	Female	EBOV	Scheduled, day 5	1
080 **	Female	EBOV	Scheduled, day 5	0
086 **	Male	EBOV	Scheduled, day 5	1
024 *	Male	EBOV	Scheduled, day 6	1
032 *	Male	Mock, PBS	Scheduled, day 6	0
034 *	Female	Mock, PBS	Scheduled, day 6	0
035 *	Female	EBOV	Scheduled, day 6	1
083 **	Female	EBOV	Scheduled, day 6	0
084 **	Female	Mock, PBS	Scheduled, day 6	1
085 **	Male	EBOV	Scheduled, day 6	0
089 **	Male	Mock, PBS	Scheduled, day 6	0
029 *	Female	EBOV	Unscheduled, day 7	27
079 **	Female	EBOV	Unscheduled, day 7	FDIC
025 **	Male	EBOV	Unscheduled, day 9	16
023 *	Male	EBOV	Unscheduled, day 10	FDIC

* Iteration 1; ** Iteration 2; EBOV–Ebola virus; FDIC–found dead in the cage.

**Table 3:** Serum viral load measured by plaque assay (PFU/mL).

	Animal ID	Day 0	Day 3	Day 4	Day 5	Day 6	Day 7	Day 9	Day of Death
Scheduled	**028**	UD	UD	N/A	N/A	N/A	N/A	N/A	3
	**031**	UD	UD	N/A	N/A	N/A	N/A	N/A	3
	**081**	UD	UD	N/A	N/A	N/A	N/A	N/A	3
	**088**	UD	UD	N/A	N/A	N/A	N/A	N/A	3
	**027**	UD	UD	2.25 × 10^1^	N/A	N/A	N/A	N/A	4
	**030**	UD	UD	1.65 × 10^2^	N/A	N/A	N/A	N/A	4
	**082**	UD	UD	5.50 × 10^1^	N/A	N/A	N/A	N/A	4
	**087**	UD	UD	2.23 × 10^2^	N/A	N/A	N/A	N/A	4
	**026**	UD	UD	1.80 × 10^2^	1.25 × 10^4^	N/A	N/A	N/A	5
	**033**	UD	1.91 × 10^3^	2.04 × 10^6^	2.36 × 10^7^	N/A	N/A	N/A	5
	**080**	UD	UD	UD	1.53 × 10^2^	N/A	N/A	N/A	5
	**086**	UD	5.50 × 10^1^	1.54 × 10^4^	1.48 × 10^7^	N/A	N/A	N/A	5
	**024**	UD	UD	UD	9.63 × 10^3^	2.28 × 10^5^	N/A	N/A	6
	**035**	UD	UD	1.40 × 10^2^	3.43 × 10^5^	3.00 × 10^6^	N/A	N/A	6
	**083**	UD	UD	UD	1.29 × 10^4^	6.88 × 10^4^	N/A	N/A	6
	**085**	UD	UD	UD	1.30 × 10^4^	2.51 × 10^5^	N/A	N/A	6
Unscheduled	**029**	UD	UD	1.53 × 10^4^	1.65 × 10^6^	3.75 × 10^6^	3.70 × 10^6^	N/A	7
	**079**	UD	UD	1.40 × 10^3^	9.00 × 10^6^	1.26 × 10^7^	N/A	N/A	7 (FDIC)
	**025**	UD	UD	UD		2.88 × 10^6^	N/A	3.60 × 10^5^	9
	**023**	UD	UD	2.25 × 10^1^	9.00 × 10^3^	4.75 × 10^5^	N/A	N/A	10 (FDIC)

N/A—not applicable; UD—undetermined, below limit of detection (25 PFU/mL); —virus detected but not quantifiable due to inconsistent data across dilution series.

**Table 4:** Summary of viral load in lymph nodes.

		Plaque Forming Units per Gram of Tissue				Genome Equivalents per Microgram of RNA				
	ID	Axillary	Mediastinal	Right Inguinal	Mesenteric	Axillary	Mediastinal	Right Inguinal	Mesenteric	Day of Death
Scheduled Euthanasia	**028**	3.75 × 10^2^	UD	1.43 × 10^2^	UD	1.50 × 10^3^	UD	6.70	6.69 × 10^1^	3
	**031**	UD	UD	UD	UD	4.65 × 10^1^	UD	6.73 × 10^1^	UD	3
	**081**	UD	UD	UD	UD	1.02 × 10^6^	2.61 × 10^1^	4.28 × 10^1^	UD	3
	**088**	4.50 × 10^4^	UD	UD	UD	9.98 × 10^3^	4.86 × 10^1^	UD	UD	3
	**027**	4.38 × 10^7^	3.88 × 10^2^	3.51 × 10^2^	UD	1.23 × 10^8^	2.63 × 10^3^	3.82 × 10^2^	5.56 × 10^2^	4
	**030**	UD	2.62 × 10^3^	8.16 × 10^2^	2.34 × 10^3^	9.85 × 10^2^	3.79 × 10^2^	5.12 × 10^1^	2.00 × 10^2^	4
	**082**	2.50 × 10^3^	UD	UD	UD	4.52 × 10^6^	7.95 × 10^1^	UD	1.89 × 10^2^	4
	**087**	1.61 × 10^3^	1.30 × 10^2^	1.25 × 10^3^	1.64 × 10^2^	2.01 × 10^4^	1.35 × 10^3^	1.44 × 10^2^	8.05 × 10^2^	4
	**026**	1.54 × 10^6^	3.93 × 10^5^	3.21 × 10^6^	8.41 × 10^5^	1.74 × 10^6^	5.00 × 10^4^	8.53 × 10^3^	1.76 × 10^4^	5
	**033**	1.40 × 10^7^	1.78 × 10^7^	2.14 × 10^6^	5.04 × 10^8^	1.51 × 10^6^	6.65 × 10^4^	5.81 × 10^5^	9.78 × 10^5^	5
	**080**	UD	1.06 × 10^3^	1.02 × 10^2^	UD	1.56 × 10^6^	4.42 × 10^2^	3.82 × 10^2^	5.44 × 10^3^	5
	**086**	9.50 × 10^6^	1.04 × 10^7^	9.94 × 10^6^	9.64 × 10^6^	1.80 × 10^8^	1.90 × 10^5^	1.16 × 10^5^	3.61 × 10^5^	5
	**024**	2.95 × 10^7^	2.28 × 10^5^	1.61 × 10^6^	1.90 × 10^4^	6.55 × 10^4^	2.12 × 10^5^	6.65 × 10^5^	1.36 × 10^6^	6
	**035**	2.35 × 10^6^	8.70 × 10^6^	1.91 × 10^6^	2.33 × 10^6^	5.44 × 10^5^	1.18 × 10^6^	6.11 × 10^4^	1.42 × 10^6^	6
	**083**	7.13 × 10^5^	1.35 × 10^7^	3.48 × 10^3^	7.39 × 10^6^	3.09 × 10^5^	7.35 × 10^5^	9.37 × 10^3^	1.02 × 10^6^	6
	**085**	1.60 × 10^7^	8.96 × 10^7^	2.68 × 10^4^	2.32 × 10^6^	2.38 × 10^4^	1.08 × 10^6^	1.65 × 10^6^	4.65 × 10^5^	6
Unscheduled	**029**	2.30 × 10^7^	5.44 × 10^6^	3.11 × 10^7^	9.61 × 10^7^	4.01 × 10^6^	2.25 × 10^6^	2.49 × 10^8^	3.49 × 10^6^	7
	**079**	6.19 × 10^6^	1.28 × 10^7^	2.54 × 10^7^	9.62 × 10^5^	7.01 × 10^3^	4.56 × 10^5^	2.11 × 10^5^	4.08 × 10^5^	7 (FDIC)
	**025**	2.33 × 10^6^	2.96 × 10^6^	1.06 × 10^6^	2.10 × 10^6^	4.39 × 10^5^	9.01 × 10^4^	7.24 × 10^4^	5.91 × 10^4^	9
	**023**	8.13 × 10^6^	2.70 × 10^6^	3.15 × 10^6^	1.31 × 10^7^	7.10 × 10^5^	2.71 × 10^6^	1.22 × 10^5^	9.29 × 10^5^	10 (FDIC)

UD—undetermined, below limit of detection; assay detection limits—25 PFU/g and 10 GE/µg of RNA.

**Table 5:** Summary of viral load in gastrointestinal tract.

		Plaque Forming Units per Gram of Tissue						Genome Equivalents per Microgram of RNA						
	ID	Stomach	Duo-Denum	Jeju-Num	Ileum	Colon	Rectum	Stomach	Duo-Denum	Jejunum	Ileum	Colon	Rectum	Day of Death
Scheduled Euthanasia	**028**	UD	UD	UD	2.04 × 10^2^	1.43 × 10^3^	UD	UD	UD	UD	UD	UD	UD	3
	**031**	UD	UD	UD	5.63 × 10^4^	4.06 × 10^3^	1.13 × 10^3^	UD	UD	1.04 × 10^1^	UD	2.97 × 10^1^	1.51 × 10^1^	3
	**081**	UD	UD	UD	UD	UD	UD	UD	UD	2.02 × 10^1^	UD	UD	UD	3
	**088**	UD	UD	UD	UD	UD	UD	UD	UD	UD	UD	UD	UD	3
	**027**	UD	UD	UD	UD	UD	UD	4.34 × 10^1^	UD	5.34 × 10^1^	UD	3.41 × 10^1^	2.35 × 10^1^	4
	**030**	UD	UD	UD	1.50 × 10^2^	1.30 × 10^1^	UD	1.32 × 10^1^	5.47 × 10^1^	7.08	1.10 × 10^2^	2.20 × 10^1^	8.05 × 10^1^	4
	**082**	UD	UD	UD	UD	UD	UD	UD	1.10 × 10^1^	UD	7.55	3.17 × 10^1^	3.59 × 10^1^	4
	**087**	UD	UD	UD	UD	1.66 × 10^2^	UD	1.12 × 10^1^	UD	UD	2.63 × 10^1^	1.34 × 10^1^	UD	4
	**026**	UD	UD	1.37 × 10^4^	2.76 × 10^3^	1.16 × 10^4^	3.07 × 10^4^	UD	2.79 × 10^2^	5.91 × 10^2^	1.97 × 10^3^	1.12 × 10^2^	1.86 × 10^1^	5
	**033**	4.80 × 10^6^	4.93 × 10^6^	1.87 × 10^6^	3.24 × 10^6^	1.11 × 10^7^	3.28 × 10^6^	UD	7.98 × 10^3^	1.14 × 10^4^	6.73 × 10^4^	8.78 × 10^4^	UD	5
	**080**	UD	UD	UD	UD	UD	UD	UD	3.80 × 10^2^	UD	UD	6.80 × 10^1^	UD	5
	**086**	5.68 × 10^5^	4.68 × 10^4^	5.74 × 10^5^	8.19 × 10^5^	8.16 × 10^5^	3.09 × 10^5^	UD	3.76 × 10^3^	UD	2.74 × 10^3^	9.67 × 10^2^	1.18 × 10^3^	5
	**024**	8.26 × 10^2^	UD	2.78 × 10^2^	6.58 × 10^1^	4.49 × 10^4^	6.47 × 10^4^	UD	1.08 × 10^6^	1.22 × 10^4^	4.48 × 10^4^	2.29 × 10^3^	1.18 × 10^1^	6
	**035**	4.99 × 10^4^	7.48 × 10^5^	5.76 × 10^5^	3.53 × 10^5^	1.31 × 10^5^	1.40 × 10^5^	1.02 × 10^3^	1.54 × 10^4^	3.01 × 10^4^	1.03 × 10^5^	1.62 × 10^4^	7.51 × 10^2^	6
	**083**	1.49 × 10^4^	8.76 × 10^4^	6.16 × 10^4^	3.94 × 10^5^	4.12 × 10^6^	1.91 × 10^6^	2.72 × 10^2^	1.07 × 10^3^	7.18 × 10^3^	6.41 × 10^4^	2.33 × 10^4^	3.41 × 10^3^	6
	**085**	4.66 × 10^5^	6.56 × 10^5^	2.85 × 10^4^	6.08 × 10^4^	5.86 × 10^5^	3.30 × 10^4^	UD	3.15 × 10^3^	9.51 × 10^2^	1.52 × 10^3^	4.88 × 10^2^	UD	6
Unscheduled	**029**	2.93 × 10^6^	4.27 × 10^6^	2.32 × 10^7^	1.16 × 10^8^	5.26 × 10^7^	3.00 × 10^7^	UD	2.63 × 10^2^	5.80 × 10^5^	1.28 × 10^6^	1.32 × 10^4^	1.48 × 10^5^	7
	**079**	1.92 × 10^5^	6.22 × 10^5^	6.96 × 10^5^	1.58 × 10^6^	1.57 × 10^6^	4.9 × 10^5^	UD	UD	5.87 × 10^3^	5.45 × 10^4^	5.53 × 10^4^	6.93 × 10^3^	7 (FDIC)
	**025**	3.18 × 10^4^	1.98 × 10^6^	1.13 × 10^5^	1.38 × 10^6^	8.38 × 10^5^	8.6 × 10^6^	UD	2.48 × 10^4^	1.58 × 10^5^	4.17 × 10^4^	4.88 × 10^3^	6.12 × 10^3^	9
	**023**	1.47 × 10^6^	1.39 × 10^7^	6.27 × 10^6^	6.58 × 10^6^	7.30 × 10^6^	8.7 × 10^7^	3.70 × 10^2^	2.41 × 10^5^	3.09 × 10^5^	2.69 × 10^5^	3.14 × 10^4^	2.01 × 10^4^	10 (FDIC)

UD—undetermined, below limit of detection; assay detection limits—25 PFU/g and 10 GE/µg of RNA.

**Table 6:** Descriptive statistics for time to onset (days) for select parameters measured in EBOV exposed nonhuman primates (NHPs).

Parameter	Number of Onsets [*n* = 20 NHP]	Mean	Standard Deviation	Min.	Max.
sCD40L	16	3.50	0.52	3.0	4.0
qRT-PCR	14	3.71	0.61	3.0	5.0
Granulocyte Counts	17	3.76	0.56	3.0	5.0
sGP	16	3.81	0.40	3.0	4.0
Telemetry Body Temperature	13	4.2	0.45	3.53	5.12
Plaque Assay Titers	16	4.25	0.77	3.0	6.0
Lymphocyte Counts	10	4.30	0.67	3.0	5.0
D-Dimer	13	4.31	1.11	3.0	6.0
Platelet Counts	6	4.50	1.38	3.0	6.0
RectalTemperature	8	4.50	0.53	4.0	5.0
aPTT	9	4.56	2.01	3.0	9.0
GGT	14	4.64	1.01	3.0	6.0
ALB	13	4.77	0.93	3.0	6.0
Red Blood Cell Counts	11	4.82	1.08	3.0	6.0
PTT	8	5.00	1.85	3.0	9.0
BUN	11	5.18	1.60	3.0	9.0

## Data Availability

The data supporting the findings of this study are available within the article and its supplementary materials.

## Supplementary Materials

The following are available online at https://www.mdpi.com/2076-2607/9/3/489/s1, Figure S1: Cell Blood Counts, Figure S2: Clinical Chemistry Parameters, Table S1: Serum Viral Load Measured by qRT-PCR Assay, targeting Glycoprotein region (genome equivalents/mL), Table S2: Summary of Viral Load in Brain, Heart, Lung, and Exposure Site; Table S3: Summary of Viral Load in Spleen, Liver, Kidney, and Adrenal Gland, Table S4: Individual Animal Gross Necropsy Findings.
