# Supplementary material for: Development of a Well-Characterized Rhesus Macaque Model of Ebola Virus Disease for Support of Product Development
Source: Microorganisms. 2021 Feb 26;9(3):489. doi: 10.3390/microorganisms9030489 (PMC7996724; doi:10.3390/microorganisms9030489)
Supplement: Supplementary file 1 [file microorganisms-09-00489-s001.zip › Supplementary Figures and Tables/Alfson_Goez-Gazi_Figure S1.pdf]

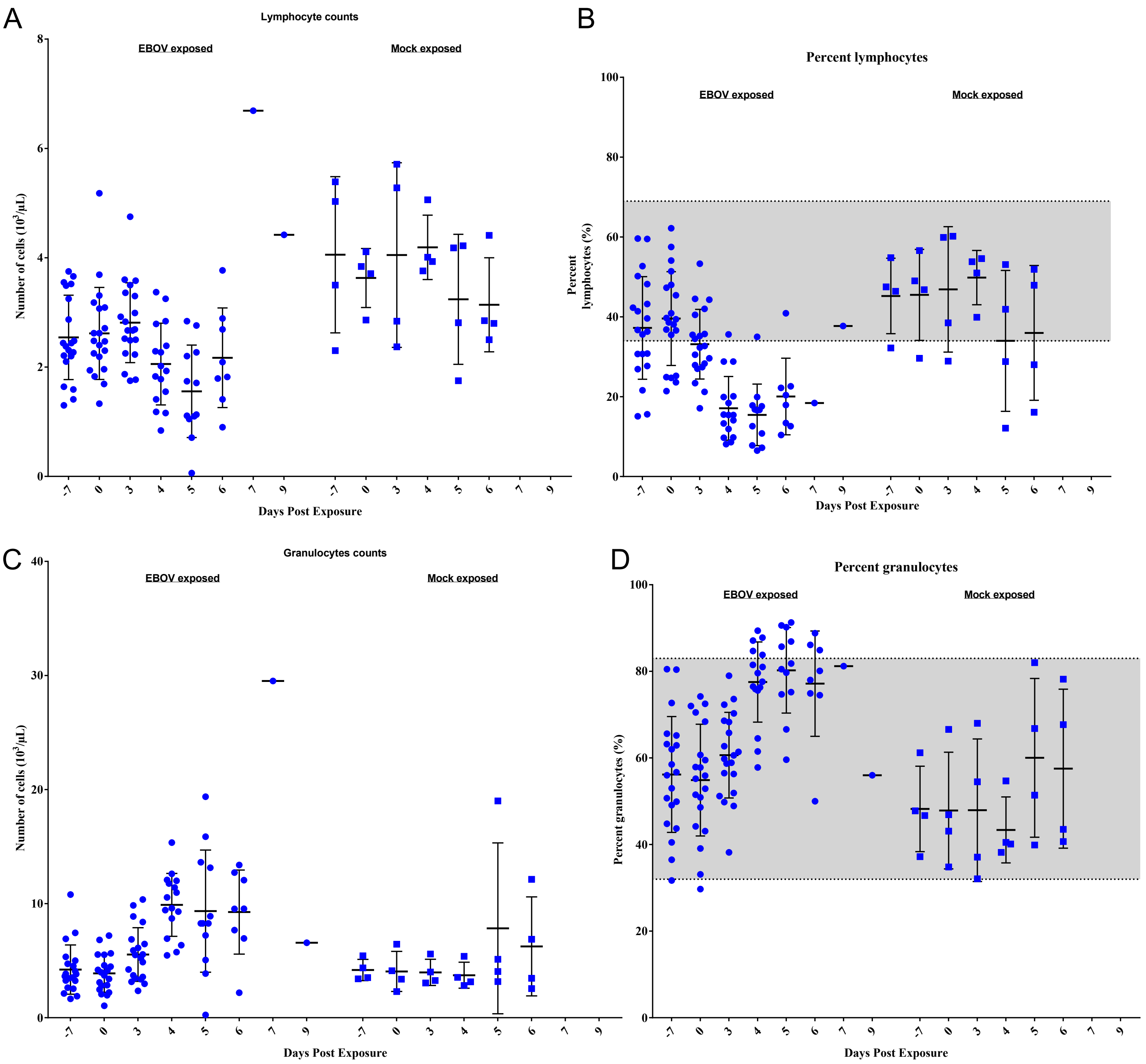

Figure S1. Cell Blood Counts in rhesus macaques intramuscularly exposed to EBOV or mock exposed with PBS. Blood was collected at pre-determined timepoints and prior to any euthanasia to determine the presence of changes in blood cells that correlate with disease. Bars represent mean and standard deviation. Grey shading represents reference range. A) Lymphocyte Counts, B) Lymphocyte Percentage, C) Granulocyte Count, and D) Granulocyte Percentage.
