# Supplementary material for: Development of a Well-Characterized Rhesus Macaque Model of Ebola Virus Disease for Support of Product Development
Source: Microorganisms. 2021 Feb 26;9(3):489. doi: 10.3390/microorganisms9030489 (PMC7996724; doi:10.3390/microorganisms9030489)
Supplement: Supplementary file 1 [file microorganisms-09-00489-s001.zip › Supplementary Figures and Tables/Alfson_Goez-Gazi_Figure S2.pdf]

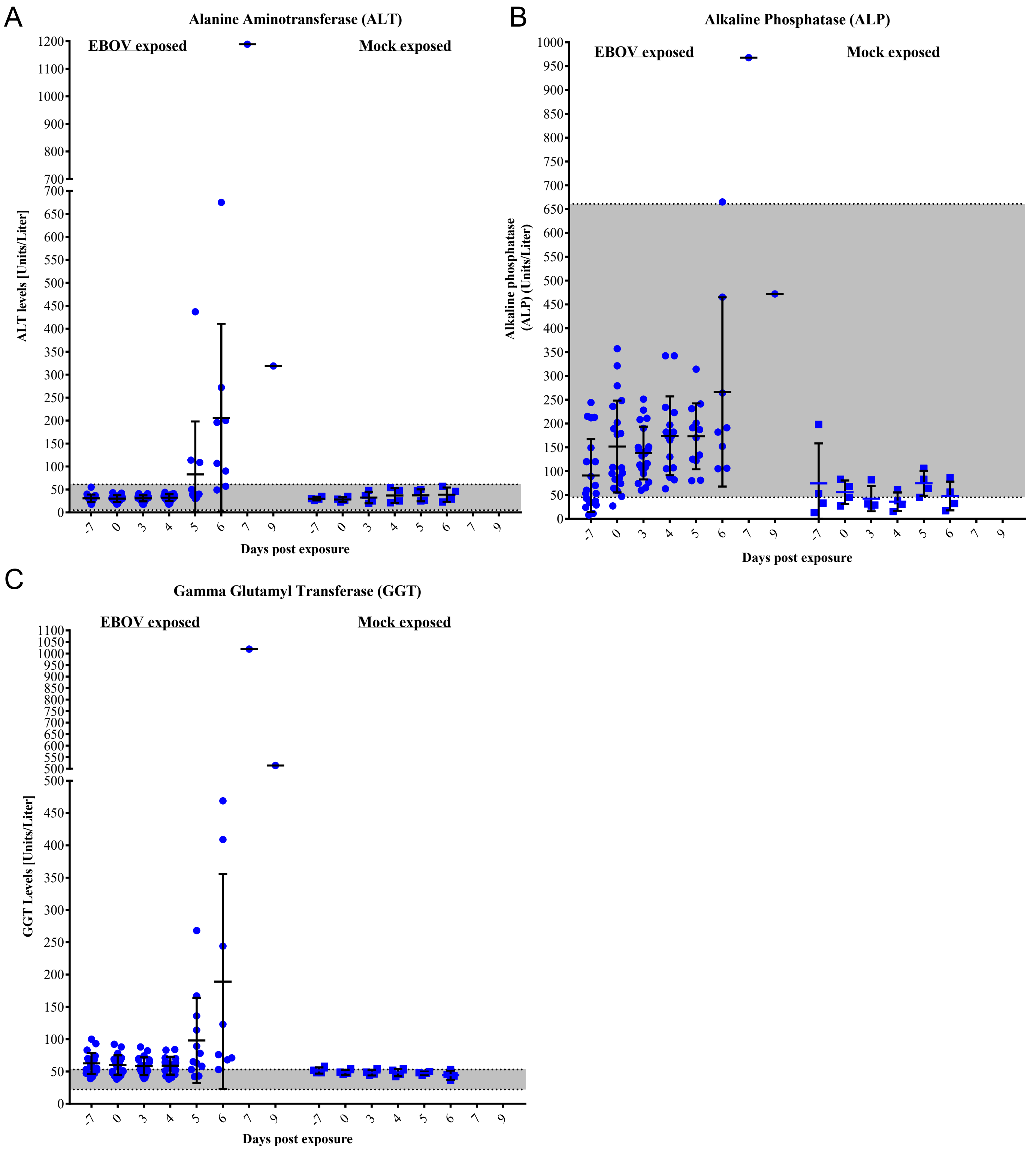

Figure S2. Clinical Chemistry Parameters in rhesus macaques intramuscularly exposed to EBOV or mock exposed with PBS. Blood was collected at pre-determined timepoints and prior to any euthanasia to determine the presence of biochemical markers that correlate with clinical pathology (hepatic function). Bars represent mean and standard deviation. Grey shading represents reference range. A) alanine aminotransferase, B) alkaline phosphatase, C) gamma-glutamyl transferase.
