# Supplementary material for: Development of a Well-Characterized Rhesus Macaque Model of Ebola Virus Disease for Support of Product Development
Source: Microorganisms. 2021 Feb 26;9(3):489. doi: 10.3390/microorganisms9030489 (PMC7996724; doi:10.3390/microorganisms9030489)
Supplement: Supplementary file 1 [file microorganisms-09-00489-s001.zip › Supplementary Figures and Tables/Alfson_Goez-Gazi_Table S4.pdf]

Table S4. Individual Animal Gross Necropsy Findings

		SE, Day 3				SE, Day 4				SE, Day 5				SE, Day 6				US (Days 7 to 9)			
**Tissue**	**Description**	**028**	**031**	**081**	**088**	**027**	**030**	**082**	**087**	**026**	**033**	**080**	**086**	**024**	**035**	**083**	**085**	**023**	**025**	**029**	**79**
**Skin**	*Petechial rash*										X		X	X	X	X	X	X	X		X
**Challenge site**	*Rash*									X					X			X			
**Inguinal LN**	*Enlarged*													X	X			X	X		X
	Dark	X	X		X	X				X	X			X						X	
	Firm		X	X	X	X	X			X	X			X	X	X		X	X	X	
**Axillary LN**	*Enlarged*							X	X	X		X	X	X	X	X	X	X	X	X	X
	Dark	X	X		X	X				X	X			X						X	
	Firm	X	X		X	X	X			X	X			X	X		X	X	X	X	
**Mediastinal LN**	*Firm*				X	X					X			X		X		X	X	X	
	Enlarged													X		X		X			
**Spleen**	*Enlarged*									X	X		X		X	X		X	X	X	X
	Firm				X		X			X					X	X	X	X	X	X	X
	Rounded				X		X			X					X	X			X	X	X
**Liver**	*Pale*										X			X	X				X	X	X
	Friable																	X			X
**Adrenal gland**	*Enlarged*																	X		X	
**Kidney**	*Red*																	X	X		
**Mesenteric LN**	*Firm*															X					X
	Enlarged									X					X				X		
**GI tract**	*Red mucosa or red foci, stomach*						X											X			
	Red mucosa, duodenum												X	X				X			
	Red mucosa, colon																		X	X	X
	Blood in lumen, rectum		X				X			X	X							X	X	X	X
**Bladder**	*Petechia or red mucosa*									X								X		X	
**Testicle**	*Blood*		NA	NA			NA	NA			NA	NA			NA	NA		X	X	NA	NA
**Uterus**	*Blood in lumen*	NA			NA	NA			NA	NA			NA	NA			NA	NA	NA	X	X

SE: Scheduled euthanasia; US: unscheduled death; GI: Gastrointestinal; X: finding was present at time of necropsy; NA: not applicable, organ not present due to animal sex.
