# Supplementary material for: Development of a Well-Characterized Rhesus Macaque Model of Ebola Virus Disease for Support of Product Development
Source: Microorganisms. 2021 Feb 26;9(3):489. doi: 10.3390/microorganisms9030489 (PMC7996724; doi:10.3390/microorganisms9030489)
Supplement: Supplementary file 1 [file microorganisms-09-00489-s001.zip › Supplementary Figures and Tables/Alfson_Goez-Gazi_Table S2.pdf]

Table S2. Summary of Viral Load in Spleen, Liver, Kidney, and Adrenal Gland

		Plaque forming units per gram of tissue				Genome Equivalents per microgram of RNA				
	**ID**	**Spleen**	**Liver**	**Kidney**	**Adrenal Gland**	**Spleen**	**Liver**	**Kidney**	**Adrenal Gland**	**Day of Death**
Scheduled Euthanasia	**028**	7.38×10^{2}	3.00×10^{2}	UD	UD	1.38×10^{3}	2.47×10^{2}	1.78×10^{1}	2.48×10^{1}	3
	**031**	1.25×10^{2}	UD	UD	UD	2.24×10^{3}	6.82×10^{2}	4.04×10^{1}	1.43×10^{1}	3
	**081**	4.00×10^{2}	UD	UD	UD	7.83×10^{2}	1.08×10^{2}	2.00×10^{2}	2.03×10^{2}	3
	**088**	UD	UD	UD	UD	9.77×10^{1}	2.12×10^{1}	UD	1.74×10^{1}	3
	**027**	4.79×10^{4}	3.00×10^{3}	UD	UD	9.28×10^{3}	2.10×10^{3}	3.76×10^{1}	8.38×10^{1}	4
	**030**	1.26×10^{6}	5.88×10^{4}	1.03×10^{2}	3.75×10^{2}	1.23×10^{5}	1.09×10^{5}	2.16×10^{2}	3.08×10^{3}	4
	**082**	5.06×10^{4}	UD	UD	UD	4.88×10^{4}	4.59×10^{3}	1.75×10^{2}	2.22×10^{2}	4
	**087**	8.46×10^{5}	5.25×10^{5}	UD¥	2.25×10^{4}	1.04×10^{6}	1.49×10^{5}	7.30×10^{2}	1.05×10^{4}	4
	**026**	6.79×10^{6}	6.88×10^{6}	7.68×10^{2}	7.50×10^{4}	1.32×10^{6}	2.05×10^{5}	1.40×10^{3}	3.43×10^{4}	5
	**033**	5.63E+08	3.69×10^{7}	3.52×10^{6}	1.11×10^{7}	7.23×10^{6}	9.68×10^{7}	2.08×10^{5}	1.45×10^{6}	5
	**080**	3.13×10^{6}	5.38×10^{5}	UD	UD	6.61×10^{5}	2.41×10^{5}	6.27×10^{2}	1.84×10^{3}	5
	**086**	8.83×10^{7}	2.17×10^{7}	6.37×10^{5}	1.34×10^{6}	3.72×10^{6}	2.74×10^{6}	2.37×10^{5}	6.30×10^{5}	5
	**024**	5.00×10^{7}	8.16×10^{6}	UD	1.64×10^{6}	3.29×10^{6}	6.42×10^{6}	2.54×10^{4}	2.28×10^{5}	6
	**035**	1.50×10^{7}	1.62×10^{7}	9.46×10^{5}	1.09×10^{6}	2.96×10^{6}	2.90×10^{6}	5.58×10^{4}	5.15×10^{5}	6
	**083**	6.06×10^{7}	5.56×10^{6}	8.99×10^{4}	9.58×10^{5}	1.60×10^{6}	2.82×10^{6}	1.89×10^{4}	2.59×10^{5}	6
	**085**	8.06×10^{6}	8.04×10^{6}	5.95×10^{4}	8.00×10^{5}	7.33×10^{6}	1.95×10^{6}	1.54×10^{4}	3.23×10^{5}	6
Unscheduled	**029**	6.88×10^{7}	6.21×10^{7}	8.12×10^{3}	5.71×10^{6}	1.70×10^{8}	1.21×10^{8}	1.26×10^{5}	2.51×10^{6}	7
	**079**	1.79×10^{7}	4.75×10^{7}	4.96×10^{6}	4.67×10^{5}	1.61×10^{8}	1.51×10^{8}	3.53×10^{5}	2.59×10^{6}	7 (FDIC)
	**025**	9.63×10^{6}	7.00×10^{6}	2.73×10^{6}	6.81×10^{5}	3.47×10^{6}	2.81×10^{5}	1.25×10^{5}	2.09×10^{5}	9
	**023**	3.83×10^{6}	1.94×10^{7}	8.78×10^{6}	3.28×10^{6}	8.31×10^{7}	1.42×10^{6}	1.74×10^{6}	2.83×10^{6}	10 (FDIC)

UD – Undetermined; Assay detection limits – 25 PFU/g and 10 GE/μg of RNA.
