# Supplementary material for: Development of a Well-Characterized Rhesus Macaque Model of Ebola Virus Disease for Support of Product Development
Source: Microorganisms. 2021 Feb 26;9(3):489. doi: 10.3390/microorganisms9030489 (PMC7996724; doi:10.3390/microorganisms9030489)
Supplement: Supplementary file 1 [file microorganisms-09-00489-s001.zip › Supplementary Figures and Tables/Alfson_Goez-Gazi_Table S3.pdf]

Table S3. Summary of Viral Load in Brain, Heart, Lung, and Exposure Site

		Plaque forming units per gram of tissue				Genome Equivalents per microgram of RNA				
	**ID**	**Brain**	**Heart**	**Lung**	**Exposure site (skin)**	**Brain**	**Heart**	**Lung**	**Exposure site (skin)**	**Day of Death**
Scheduled Euthanasia	**028**	UD	UD	2.50×10^{1}	1.62×10^{3}	UD	4.02×10^{1}	UD	1.02×10^{2}	3
	**031**	UD	UD	UD	UD	1.60×10^{1}	UD	UD	3.53×10^{5}	3
	**081**	UD	UD	UD	UD	2.43×10^{1}	5.83×10^{1}	UD	1.84×10^{2}	3
	**088**	UD	UD	UD	UD	1.38×10^{1}	8.60×10^{1}	UD	9.83×10^{2}	3
	**027**	UD	3.05×10^{1}	7.08×10^{2}	1.35×10^{3}	UD	4.54×10^{2}	UD	1.38×10^{3}	4
	**030**	UD	UD	UD	8.27×10^{1}	9.38×10^{2}	7.67×10^{2}	UD	UD	4
	**082**	UD	UD	UD	UD	1.82×10^{1}	7.86×10^{1}	2.14×10^{1}	6.09×10^{1}	4
	**087**	UD	UD	2.42×10^{2}	7.95×10^{1}	2.60×10^{1}	5.27×10^{2}	UD	1.81×10^{1}	4
	**026**	1.95×10^{2}	5.37×10^{3}	2.69×10^{4}	1.03×10^{6}	1.83×10^{2}	1.63×10^{3}	UD	4.05×10^{6}	5
	**033**	6.85×10^{4}	4.72×10^{5}	1.13×10^{7}	1.50×10^{7}	7.25×10^{3}	7.18×10^{5}	UD	7.89×10^{6}	5
	**080**	UD	2.66×10^{1}	UD	UD	$3.48 \text{ X } 10^{1}$	1.03×10^{2}	UD	1.69×10^{1}	5
	**086**	4.77×10^{4}	3.24×10^{5}	1.50×10^{6}	3.93×10^{6}	1.05×10^{4}	1.42×10^{5}	UD	1.02×10^{6}	5
	**024**	UD	4.25×10^{4}	6.71×10^{5}	8.99×10^{5}	2.54×10^{3}	5.66×10^{4}	UD	1.72×10^{4}	6
	**035**	1.51×10^{4}	9.08×10^{7}	1.52×10^{6}	1.10×10^{7}	1.41×10^{4}	6.37×10^{4}	UD	8.80×10^{6}	6
	**083**	9.84×10^{2}	9.96×10^{4}	2.50×10^{5}	2.41×10^{3}	1.01×10^{3}	9.93×10^{3}	3.60×10^{2}	8.38×10^{5}	6
	**085**	4.83×10^{2}	7.16×10^{4}	1.42×10^{5}	9.35×10^{1}	8.01×10^{2}	1.93×10^{4}	UD	1.42×10^{3}	6
Unscheduled	**029**	2.51×10^{6}	9.70×10^{5}	3.56×10^{7}	9.80×10^{5}	2.29×10^{5}	4.18×10^{5}	UD	2.93×10^{4}	7
	**079**	7.50×10^{5}	3.36×10^{6}	1.17×10^{7}	1.29×10^{5}	3.12×10^{5}	1.01×10^{5}	3.02×10^{1}	8.99×10^{3}	7 (FDIC)
	**025**	4.76×10^{4}	7.78×10^{4}	1.23×10^{6}	2.10×10^{6}	5.70×10^{3}	9.36×10^{4}	1.29×10^{3}	1.93×10^{5}	9
	**023**	7.34×10^{5}	3.78×10^{6}	7.00×10^{6}	4.62×10^{5}	2.39×10^{4}	2.02×10^{5}	UD	4.39×10^{2}	10 (FDIC)

*UD – Undetermined; Assay detection limits – 25 PFU/g and 10 GE/μg of RNA.
