# Supplementary material for: Development of a Well-Characterized Rhesus Macaque Model of Ebola Virus Disease for Support of Product Development
Source: Microorganisms. 2021 Feb 26;9(3):489. doi: 10.3390/microorganisms9030489 (PMC7996724; doi:10.3390/microorganisms9030489)
Supplement: Supplementary file 1 [file microorganisms-09-00489-s001.zip › Supplementary Figures and Tables/Alfson_Goez-Gazi_Table S1.pdf]

Table S1. Serum Viral Load Measured by qRT-PCR Assay, targeting Glycoprotein region (genome equivalents/mL)

	Animal ID	Day 0	Day 3	Day 4	Day 5	Day 6	Day 7	Day 9	Day of Death
Scheduled Euthanasia	**028**	UD	UD	N/A	N/A	N/A	N/A	N/A	3
	**031**	UD	UD	N/A	N/A	N/A	N/A	N/A	3
	**081**	UD	UD	N/A	N/A	N/A	N/A	N/A	3
	**088**	UD	UD	N/A	N/A	N/A	N/A	N/A	3
	**027**	UD	UD	4.33×10^{3}	N/A	N/A	N/A	N/A	4
	**030**	UD	3.91×10^{3}	2.60×10^{3}	N/A	N/A	N/A	N/A	4
	**082**	UD	UD	UD	N/A	N/A	N/A	N/A	4
	**087**	UD	UD (p)	1.86×10^{4}	N/A	N/A	N/A	N/A	4
	**026**	UD	UD	6.99×10^{3}	6.84×10^{5}	N/A	N/A	N/A	5
	**033**	UD	4.93×10^{3}	7.90×10^{4}	4.74×10^{6}	N/A	N/A	N/A	5
	**080**	UD	UD (p)	UD	UD	N/A	N/A	N/A	5
	**086**	UD	1.48×10^{3}	4.30×10^{4}	1.35×10^{8}	N/A	N/A	N/A	5
	**024**	UD	UD	5.78×10^{3}	3.09×10^{6}	7.02×10^{7}	N/A	N/A	6
	**035**	UD	5.20×10^{3}	UD	2.79×10^{7}	1.83×10^{8}	N/A	N/A	6
	**083**	UD	UD	2.66×10^{3}	3.04×10^{5}	UD	N/A	N/A	6
	**085**	UD	UD	2.14×10^{3}	1.67×10^{5}	1.37×10^{7}	N/A	N/A	6
Unscheduled	**029**	UD	UD	1.05×10^{5}	1.30×10^{8}	6.08×10^{8}	3.94×10^{8}	N/A	7
	**079**	UD	1.88×10^{3}	1.17×10^{5}	1.09×10^{8}	5.43×10^{8}	N/A	N/A	7 (FDIC)
	**025**	UD	UD (p)	UD	2.32×10^{4}	2.29×10^{7}	N/A	1.21×10^{8}	9
	**023**	UD	UD	UD	2.42×10^{5}	2.69×10^{7}	N/A	N/A	10 (FDIC)

UD – Undetermined, below assay detection limit (1,000 GE/mL); N/A - not applicable, animal deceased prior time point; FDIC – found dead in cage; (p)—plasma used instead of serum due to insufficient serum availability.
